# Identification, Quantification, and Elimination of NO_*x*_ and NH_3_ Impurities for Aqueous and Li-Mediated Nitrogen Reduction Experiments

**DOI:** 10.1021/acsenergylett.3c01130

**Published:** 2023-08-02

**Authors:** Boaz Izelaar, Davide Ripepi, Dylan D. van Noordenne, Peter Jungbacker, Ruud Kortlever, Fokko M. Mulder

**Affiliations:** † Large Scale Energy Storage, Process and Energy Department, Faculty of Mechanical, Maritime and Materials Engineering, Delft University of Technology, 2628 CB Delft, The Netherlands; ‡ Materials for Energy Conversion and Storage, Chemical Engineering Department, Faculty of Applied Sciences, Delft University of Technology, 2629 HZ Delft, The Netherlands

Ammonia (NH_3_) ranks among the largest bulk chemical products in the world, with an annual production of 178 million tons and an estimated annual market growth of 3–5% to meet the global demand for fertilizer in the agricultural sector due to an increasing world population.^1,2^ The majority of NH_3_ is produced by the Haber–Bosch process, wherein elevated temperatures (300–500 °C) and pressures (200–300 bar) are required.^3^ In addition, the current process has a major environmental impact (∼1% of the global greenhouse emissions), mostly due to the production of hydrogen by steam-methane reforming.^4^ To meet the net-zero emissions goal by 2050, as established in the latest IPCC report,^5^ ammonia must be produced via a sustainable pathway.^6^ Direct electrocatalytic synthesis of ammonia from dinitrogen and water at mild conditions could potentially offer a carbon-free alternative, resilient to intermittent renewable energy generation.^7^

Despite the large research efforts on nitrogen electroreduction in aqueous electrolytes, current NH_3_ synthesis rates remain extremely low (0.003–14 nmol cm^–2^ s^–1^).^8^ This is mainly due to the lack of a suitable electrocatalyst and competition with the hydrogen evolution reaction (HER). Besides, the reliable quantification of these low ammonia yields has raised several concerns in the scientific community. The presence of trace amounts of extraneous N species (such as, NH_3_, NO_*x*_, N_2_O, NO_*x*_^–^, and other, more labile forms of N) has led to an increasing number of reported false positives and non-reproducible results.^9−13^ Overall, the electrochemical reduction of nitrogen oxide species into ammonia is more facile than the nitrogen reduction reaction (NRR) on many transition metals.^14−16^ An exception is N_2_O, which has been proven to only electroreduce into N_2_ on several transition metals.^15,17^ This implies that N_2_O is not a concerning impurity source for the NRR. Numerous rigorous experimental protocols have been proposed to perform reliable quantification of NH_3_ produced by electrochemical N_2_ reduction.^18,19^ Ultimately, purified ^15^N_2_-labeled gas is used to reliably confirm the electroreduction of ^15^N_2_ into the unambiguously traceable ^15^NH_3_.^20^ However, over recent years, a significant amount of publications, that implemented all recommended control experiments (including ^15^N_2_ gas), could not be duplicated.^21,22^ A common issue is that the efficacy of the implemented purification methods, such as gas purification or N removal from lab materials, is often poorly assessed. Additionally, it remains challenging to identify the main sources of extraneous N and to what extent it contributes to elevated NH_3_ background levels.

In this Viewpoint, we present a systematic impurity screening of the most common used lab materials and gases in the aqueous and non-aqueous lithium-mediated NRR field. Not only does this give new insights into the origin of an impurity, but it also highlights the severity of specific sources for an impurity. More importantly, the effectiveness of earlier proposed cleaning strategies for gases, cell components, materials, and lab consumables are re-evaluated and further optimized.

We discover by using sensitive *in-line* gas detection methods that ^14^N_2_ and Ar feed gases are free of NH_3_ and NO_*x*_ impurities and do not require excessive N purification. Only ^15^N_2_ is contaminated and must be purified with a certified or pre-assessed gas filter. Often-used in-house-made scrubbers or liquid traps have a much lower N trapping efficiency and should not be implemented. The accumulation of atmospheric N species on ambient exposed cell components, chemicals, lab consumables, and other labware is inevitable and is most likely the main source of elevated NH_3_ background levels. This can be significantly reduced by our recommended pre-treatment procedures. For Li-NRR systems, trace amounts of nitrate might be present in Li-salts and can interfere with the genuine NH_3_ quantification, especially at low concentrations. Therefore, we recommend to determine a nitrate background concentration since it cannot be removed from the salt. Ultimately, this work will equip the experimentalist with specific guidelines and tools to perform more reliable NRR measurements.

## Impact of Atmospheric NO_*x*_ and NH_3_ Species

**Figure 1:**
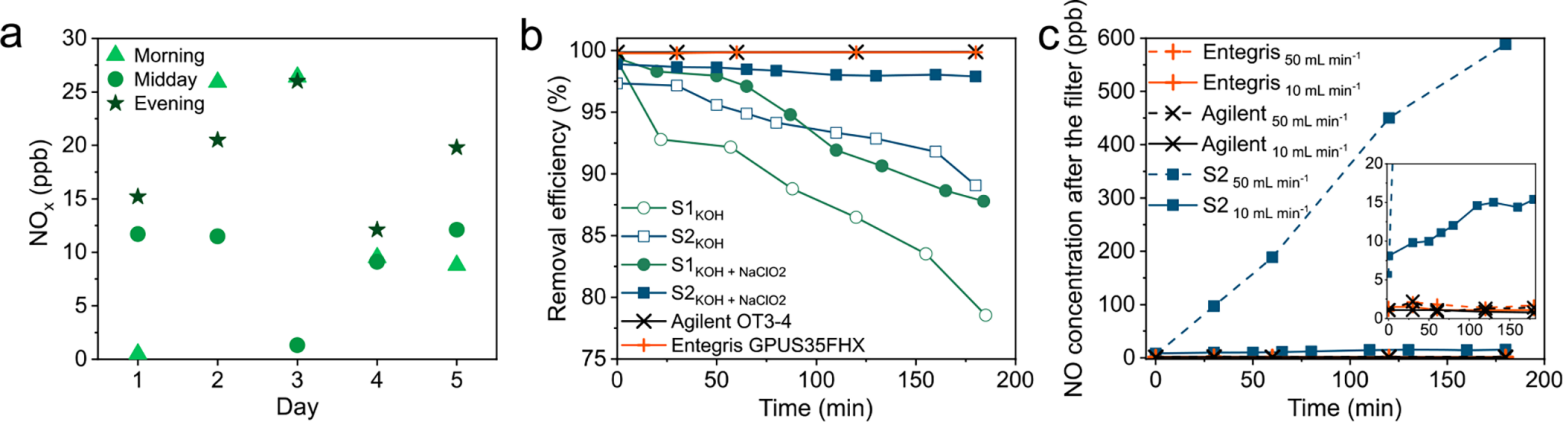
(a) Morning, midday, and evening measurements of atmospheric NO_*x*_ concentrations, recorded daily around 9.00, 12.30, and 18.00. Each data point is the average of the measured NO_*x*_ concentration over 5 min. (b) NO_*x*_ removal efficiency over time, measured for an inlet gas mixture of 50 ppm of NO in He at 10 mL min^–1^ for different scrubbers and liquid traps. S1 and S2 indicate two standard scrubbers connected in series and the in-house-made scrubber, respectively. (c) NO concentrations measured over time at the outlet of each gas filter with an inlet gas mixture of 50 ppm of NO in He at 50 (dashed line) and 10 (solid line) mL min^–1^. The in-house-made scrubber (S2) is filled with a 0.1 M KOH and 0.1 M NaClO_2_ trapping solution. The complete data set with flow rates from 1 to 50 mL min^–1^ is given in Figure S8.

One potential source of the extraneous N species can stem from the accumulation of atmospheric NH_3_ or NO_*x*_ on exposed materials. The presence of NH_3_ in the atmosphere is primarily caused by emissions from the agricultural sector, where NH_3_ volatilization occurs due to intensified herbivore production and field-applied manure.^23^ These emissions vary regionally and depend on multiple factors, such as wind direction and speed, humidity, and usage of N fertilizers. The monthly average atmospheric NH_3_ concentration in The Netherlands varies between 2 and 44 ppb,^24^ which might seem negligible. However, it is expected that long-term atmospheric exposure of chemicals, consumables, and glassware employed in NRR experiments will lead to an unavoidable introduction of contaminants due to the release of adsorbed NH_3_. The majority of atmospheric NO_*x*_ emissions are derived from industrial and automotive combustion of fossil fuels.^25^ Atmospheric NO_*x*_ concentrations in our laboratory were measured with a chemiluminescent NO_*x*_ analyzer (details available in the Supporting Information). Our results show that the concentrations fluctuated over the course of five consecutive days, with a maximum atmospheric concentration of 27 ppb (Figure 1a). However, the uptake rates during 24 h of both atmospheric NO_*x*_ and NH_3_ in water and freshly prepared 0.1 and 1 M KOH solutions were negligible (Figure S1). This indicates that short-term atmospheric exposure is not an issue. Long-term accumulation of NO_*x*_ impurities was monitored for both low- and high-purity grade KOH (85% and 99.99%), and it was found to depend solely on the storage conditions (Figure S2). KOH bottles stored in a chemical safety cabinet, hence exposed to the laboratory environment for a considerable time period (10 months), contained 4.4 μmol NO_3_^–^ L^–1^ in a freshly prepared 1 M KOH solution, while NO_2_^–^ concentrations were negligible (<0.2 μmol NO_2_^–^ L^–1^). Remarkably, storing the KOH pellets in a vacuum desiccator for approximately 9 months reduced the NO_*x*_ impurities to negligible levels (<0.3 μmol NO_3_^–^ L^–1^). Therefore, it is strongly advised to store chemicals in controlled environments such as desiccators or Ar gloveboxes.

## Impurity Assessment of the Feed Gases

Feed gases are suspected to contain ppm levels of NO_*x*_ that can be continuously introduced in the electrolyte during reactant gas saturation. We used a commercially available NO_*x*_ analyzer to assess our high-purity (99.999%) He, ^14^N_2_, and Ar gases (see Supporting Information, Figure S3). Additional effort was made to screen the gases for trace levels of NH_3_ with our recently developed gas chromatograph (GC).^26^ Our analysis reveals that the NH_3_ and NO_*x*_ impurities in all the gases are extremely low. NH_3_ concentrations do not exceed the lower detection limit (LOD_NH_3__ < 150 ppb) of the GC, and the NO_*x*_ content falls in the instrument’s LOD (1 ppb). High-purity ^14^N_2_ and Ar gases are manufactured by cryogenic distillation of air. Low concentrations (ppb level) of atmospheric NH_3_ and NO_*x*_ can end up in the process but will be separated because of their significantly higher boiling point. This justifies our observation, while it is in contradiction with earlier claims. If *in-line* gas detection methods are not available or used, it remains challenging to adequately quantify impurities in the gas stream due to interference from other sources.

Conversely, a ^15^N_2_ isotopologue is commercially available at a lower purity level than the conventional ^14^N_2_; thus it might contain a higher concentration of contaminants. As such, we measured up to 9.8 ppm of ammonia contained in a ^15^N_2_ gas bottle (99% purity, Sigma-Aldrich), as reported in Figure S4a. By using isotope-sensitive GC-MS,^27^ we found that the totality of the measured ammonia is in the form of ^15^NH_3_ (Figure S4b). The presence of ^15^NH_3_ presumably derives from traces of unreacted ^15^NH_3_ used during the catalytic oxidation process for the production of ^15^N_2_ gas from isotopically enriched ^15^NH_3_.^28^ Although not measured by us, different ^15^NO_*x*_ species were previously detected in various ^15^N_2_ gas bottles and can be derivatives from the production process (Table S1). It should be noted that measuring gaseous NH_3_ can be subject to underestimation, due to ammonia physisorption. To avoid this, it is recommended to use a direct gas analysis method in combination with inert materials for all the surfaces that are in contact with the gaseous sample. In fact, Figure S4a shows that no ammonia was detected when the same ^15^N_2_ gas was dosed via a non-passivated mass flow controller. Prolonged ^15^N_2_ bubbling into the electrolyte is often necessary to reach saturation, which means that the use of cumulative quantification methods requires several hours of reaction time to collect significant amounts of ^15^NH_3_.^27^ This issue can be partly circumvented by adopting a gas recirculation setup in combination with a suitable gas filter to save costs and minimize accumulation of impurities.^29^ From our analysis, it seems that, especially for the execution of ^15^N_2_ control experiments, the implementation of a gas purifier is strictly necessary.

## Feed Gas Purification Methods

Strategies to purify the feed gases are based on catalytic reduction or scrubbing using commercially available certified gas filters (<1 ppb),^21,30^ in-house-made catalytic filters (e.g., based on a Cu-Zn-Al oxide),^31^ or scrubbers containing a liquid trap.^32−34^ The latter are, to some extent, more economic and are therefore more common. However, it is especially important for uncertified filter systems, such as in-house-developed scrubbers or catalytic filters, to assess their N removal functionalities.

Here, the NO_*x*_ and NH_3_ removal efficiency is examined for a set of commonly used filters by purging them with 50 ppm of NO in He or 13.8 ppm of NH_3_ in ^14^N_2_ for 3 h at experimentally relevant flow rates. We first tested two standard 20 mL scrubbers with a glass frit (Supelco Analytical, 6-4835) connected in series (Figure S5). The poor solubility of NO in aqueous media results in less than 25% NO removal efficiency when using Milli-Q water (Figure S6). Alkaline solutions are a common choice because gaseous NO_*x*_ can be trapped in the form of NO_*x*_^–^.^35,36^ Substituting water with 0.1 M KOH already enhances the NO removal efficiency up to 78%.

Previous studies recommended the use of strong oxidizing agents, such as KMnO_4_ or NaClO_2_, to convert NO directly into soluble NO_2_^–^ or NO_3_^–^ and improve the overall filter performance.^8^ NaClO_2_ was mentioned as one of the most effective oxidants and is evaluated in the present work.^37^ A solution of 0.1 M NaClO_2_ in 0.1 M KOH removed 88% of NO after 3 h purging time (Figure 1b). Additionally, the scrubbing efficiency can be increased by optimizing the gas residence time and the bubble contact area between the gas–liquid interface. As such, inert polytetrafluoroethylene (PTFE) beads were inserted into a 30 cm long, 25 mL in-house-made scrubber (see Figure S7). This results in a further improvement in the removal efficiency, up to 98% over the course of 3 h at 10 mL min^–1^ (Figure 1b). However, the trapping efficiency drops drastically at higher flow rates (>10 mL min^–1^), as is illustrated in Figure 1c, which limits this purification strategy only to lower flow rates. Remarkably, the commercially certified gas filters (Agilent OT3-4 and Entegris GPUS35FHX) show a consistent unity removal efficiency, within the 1–50 mL min^–1^ range (Figure 1c and Figure S8). NH_3_ was completely eliminated by both commercial filters and our scrubber containing a 0.1 M NaClO_2_ and 0.1 M KOH solution (Figure S9), which was expected due to the high ammonia solubility in water (∼500 g L^–1^). This analysis shows that certified commercial filters are the most efficient and durable solution for feed gas purification. Furthermore, both filters have been extensively used in our laboratories for over 1 year without showing any sign of decay in performance. Moreover, they do not require extensive cleaning and preparation procedures. Lastly, commercial filters are widely accessible and affordable, often with the possibility of being conveniently regenerated via thermal H_2_ treatments.

## Screening of Lab Consumables

Besides the impurity contributions from atmospheric N species and ^15^N_2_ gas, there are additional concerns regarding lab consumables because significant NO_3_^–^ concentrations have been observed earlier.^38,39^ Therefore, we screened various consumables from our laboratory supply cabinets, including polypropylene 0.1–1 mL pipet tips, 1.5–12 mL sample tubes, and latex and nitrile chemically resistant gloves. For the analysis of the polypropylene consumables, the pipet tips and tubes were submerged and sonicated in 0.1 M KOH for 15 min. This procedure was repeated five times while reusing the same alkaline solution (more details in the Supporting Information). Remarkably, the N content per item is negligible (3–7 nmol), which was unexpected due to continuous ambient exposure. Nevertheless, several 1.5 mL sample tubes that were directly analyzed after arrival were completely free of any N impurities (Figure S10). This demonstrates that accumulation of adsorbed atmospheric N is inevitable, as was earlier observed for our KOH salts, but is to some extent less severe, and the N species can simply be removed with water.

**Figure 2:**
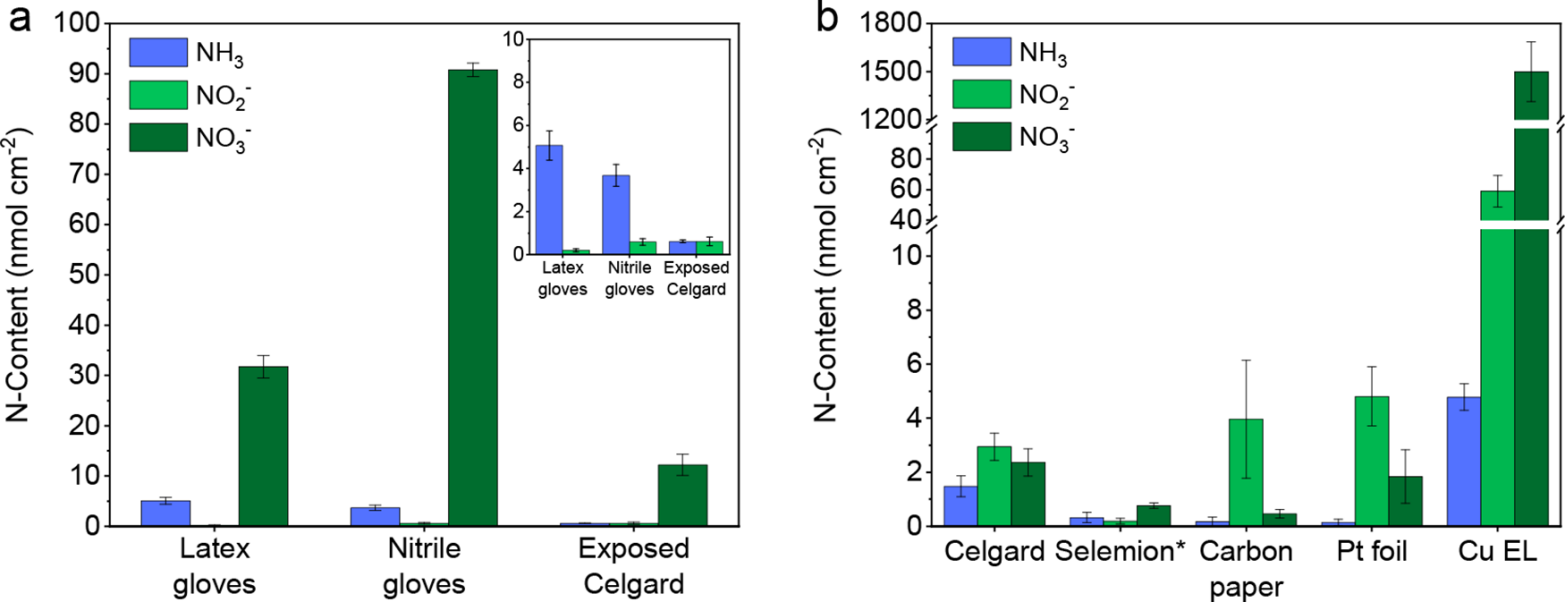
(a) NH_3_, NO_2_^–^, and NO_3_^–^ content of patches (6.0 cm × 6.0 cm) of latex and nitrile gloves cut from the center of the each glove. The N content was determined by cutting the patches into smaller chunks and sonicating them in 30 mL of 0.1 M KOH solution for 15 min. A Celgard membrane (2.5 cm × 2.5 cm) was exposed by rubbing the top and bottom surfaces with a nitrile glove. (b) The NH_3_, NO_2_^–^, and NO_3_^–^ content of cell materials was determined by sonicating 2.5 cm × 2.5 cm samples, except for the carbon paper and Cu electrodeposited on carbon paper (Cu EL), which were 1.2 cm diameter discs, in a 0.1 M KOH solution for 15 min. NH_3_ detection was done by the spectrophotometric indophenol blue method. Both NO_2_^–^ and NO_3_^–^ were quantified by ion chromatography. *NO_2_^–^ assay was performed with the spectrophotometric Griess test due to Cl^–^ overlap in the ion chromatogram. The error bars indicate the standard deviation of three individual experiments.

Patches of latex and nitrile gloves (6 cm × 6 cm) were screened by cutting the patches in little chunks and sonicating them collectively in 0.1 M KOH for 15 min. The latex gloves released reproducible quantities of 5.1 ± 0.7 nmol NH_3_ cm^–2^ and 31.7 ± 2.2 nmol NO_3_^–^ cm^–2^, while the nitrile gloves released 3.7 ± 0.5 nmol NH_3_ cm^–2^ and 90.8 ± 1.3 nmol NO_3_ cm^–2^. These significant NO_3_^–^ concentrations are most likely remaining trace impurities from the calcium nitrate used as coagulant material to harden the gloves during the manufacturing process. Not all manufacturers use calcium nitrate as a coagulant, which can explain the NO_*x*_^–^ variations reported in the literature.^19^ Regardless, direct contact with electrolyte-exposed surfaces, such as membranes, electrodes, glassware, etc., should be avoided as much as possible. To demonstrate the impact, we performed a qualitative assessment (see the Supporting Information) by rubbing a nitrile glove over the Celgard membrane and observed that reproducible amounts of N species (0.6 ± 0.1 nmol NH_3_ cm^–2^, 0.6 ± 0.2 nmol NO_2_^–^ cm^–2^, 12.2 ± 2.1 nmol NO_3_^–^ cm^–2^) were released (Figure 2a). This shows that especially NO_3_^–^ can be unintentionally introduced during cell assembly.

## Encountered Impurities in Commonly Used Cell Materials

Nafion membranes are notorious for their initial NH_4_^+^ uptake and release during NRR experiments. Here, the buildup of atmospheric NH_4_^+^ appears to be the main issue,^40^ and it remains difficult to remove because of its ion-selective and porous properties. Impurity effects in other commonly used membranes and electrode materials are, to some extent, unexplored. This motivated us to review other types of membranes, carbon paper (often used as a support), Pt foil, and a Cu electrode prepared by electrodeposition (Cu EL). A pre-defined geometrical area (indicated below) of each particular component was sonicated in 0.1 M KOH for 15 min either as received or after a treatment step for the quantification of trapped N impurities.

Celgard (3401) microporous membranes are considered cleaner alternatives to ion-exchange membranes.^20^ From our analysis, we confirm that NH_3_ levels for a 2.5 cm × 2.5 cm Celgard membrane are negligible (<1.5 nmol cm^–2^), as shown in Figure 2b. However, we found a relatively high amount of NO_*x*_^–^ species of around 7.5 nmol cm^–2^. According to the manufacturer, no sources of NO_*x*_ reactants were used during the production process, hence it is likely that physisorption of atmospheric NO_*x*_ occurred and accumulated over time. Yet, simply rinsing with water reduces impurity levels to <1 nmol cm^–2^. Anion-exchange membranes (AEMs), also commonly used in the NRR field, are mostly used with alkaline electrolytes and have the lowest ammonia crossover rates. AEM ionomers consist of positively charged quaternary ammonium functional groups that give the membrane its anion-selective properties. One could expect that, due to degradation and protonation of the N-functional groups, spontaneous ammonia formation occurs.^9,10,41^ However, we did not observe any sign of ammonia leaching from a 2.5 cm × 2.5 cm AEM (Figure 2b), even after 1 h of sonication (Figure S11). Additionally, the amount of NO_*x*_^–^ species was negligible, which is most likely related to the wetted and sealed storage of the membrane.

Catalyst and electrode materials can also be a potential source of N contaminants. Electrocatalysts prepared by using concentrated ammonia solvents or nitrate compounds should ideally be avoided. If usage is necessary, then additional pre-treatment steps and careful examination of the removal effectiveness are advised. Herein, an example is discussed where a 1.13 cm^2^ copper electrode (Cu EL) was prepared by electrodeposition using 0.5 M Cu(NO_3_)_2_ on carbon paper.^42^ From Figure 2b, it becomes clear that a freshly prepared Cu EL released enormous amounts of NO_3_^–^ (1499 ± 186 nmol cm^–2^). Left-over NO_*x*_^–^ can ideally be electroreduced with cyclic voltammetry by scanning the Cu EL between −0.2 and −0.7 V vs RHE in 0.1 M KOH (see the Supporting Information). More than 98% of the initial N-content was removed by this strategy, although the remaining ∼30 nmol is still significant (Figure S12). Alternatively, metal nitrate hydrates can be thermally decomposed into metal oxides, water, and gaseous NO_*x*_. The Cu EL was kept at 200 °C overnight because supported Cu(NO_3_)_2_ hydrate decomposition starts at 175 °C.^43^ The thermal decomposition strategy was able to remove 99.3% of the initial N-content, indicating that it is more efficient than cyclic voltammetry. Moreover, this method was applied earlier to remove NO_*x*_^–^ species from commercial metal oxide powders, and similar removal rates were reported.^12^

Platinum foil is commonly used as an anode material due to its high stability. After excessively rinsing a 2.5 cm × 2.5 cm Pt foil with H_2_O, approximately 6 nmol cm^–2^ of NO_*x*_^–^ was released. This quantity is comparable with that found with the untreated Celgard membrane, which suggests that atmospheric adsorbed NO_*x*_ species on the Pt are more stable, forming most likely Pt mononitrosyls.^44^ Flame annealing is an often used technique to remove organic impurities and to pre-oxidize the Pt surface. Interestingly, the flame annealing step provokes an increase in the N impurities (Figure S12). Sonicating the Pt foil in 0.1 M KOH or applying the thermal decomposition method was sufficient to reduce impurities to a bare minimum.

## NO_3_^–^ Assay of Common Used Lithium Salts in Li-NRR

**Figure 3:**
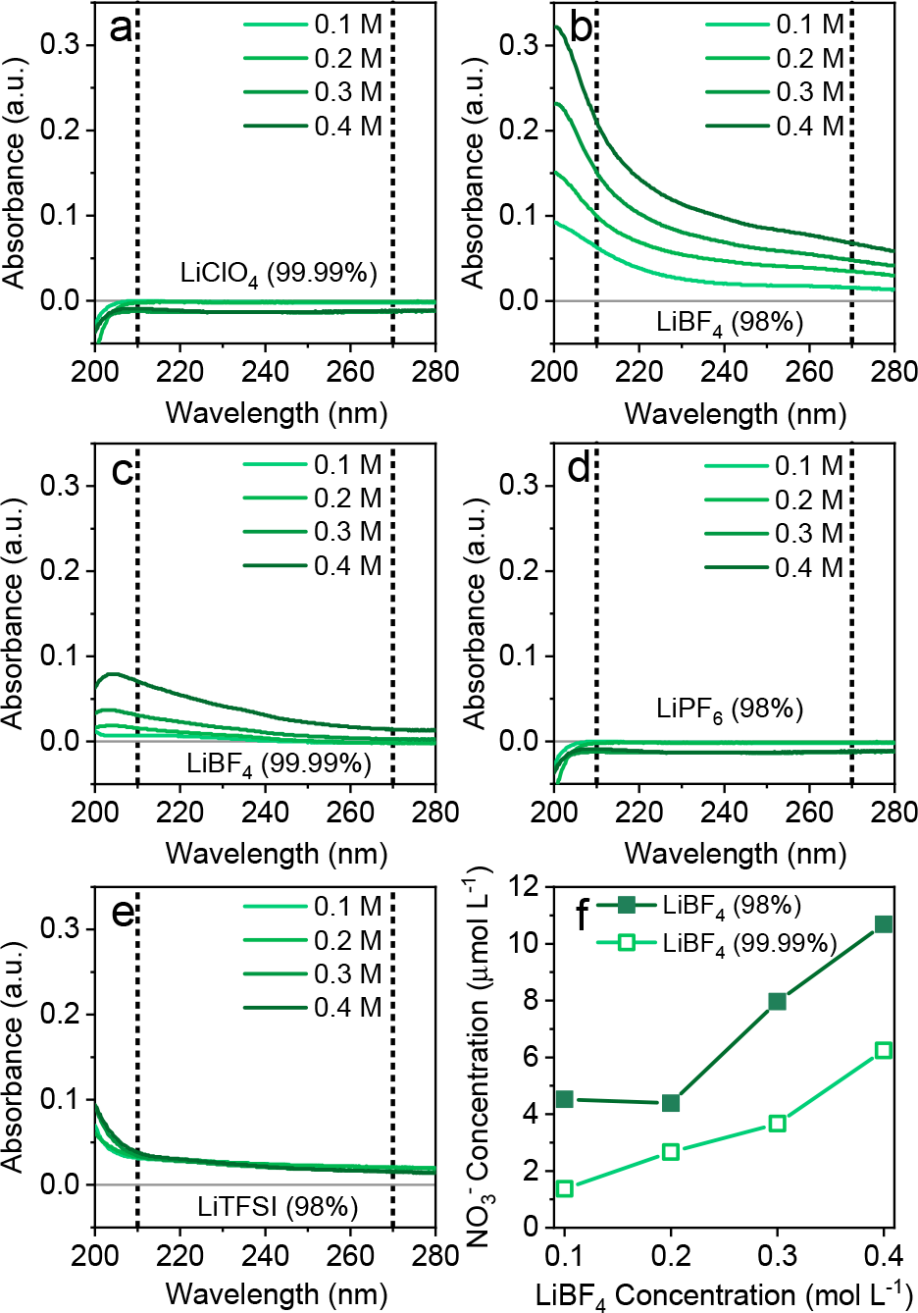
NO_3_^–^ assay showing UV spectra at different salt concentrations of (a) LiClO_4_ (99.99%, Sigma), (b) LiBF_4_ (98%, Sigma), (c) LiBF_4_ (99.99%, Sigma), (d) LiPF_6_ (98%, Honeywell), and (e) LiTFSI (98%, Sigma). (f) NO_3_^–^ concentrations as a function of the LiBF_4_ concentration.

NRR with electroplated lithium as a N_2_ activator (Li-NRR) has recently gained significant scientific interest. There are, however, various concerns about high NO_3_^–^ concentrations in Li-salts,^45^ which can easily be converted to NH_3_ in these extremely reduced environments. Herein, LiClO_4_, LiBF_4_, LiPF_6_, and lithium bis(trifluoromethanesulfonyl)imide (LiTFSI, also abbreviated as LiNTf_2_) are screened with dual-wavelength ultraviolet (UV) spectroscopy for NO_3_^–^ quantification.^46^Figure 3 shows that LiClO_4_ and LiPF_6_ are free of NO_3_^–^. Clear UV absorbance at 210 nm (associated with NO_3_^–^) was measured for LiBF_4_ and LiTFSI. Any organic interference at 210 nm was compensated by subtracting 2 times the absorbance at 270 nm (elaborated in the Supporting Information). After this correction, LiTFSI has no noteworthy NO_3_^–^ absorbance, while LiBF_4_ in Figure 3f shows a clear upward trend in NO_3_^–^ levels as a function of the salt concentration. It is important to note that NO_3_^–^ quantities can vary with different purities, suppliers, and batches.^45^ Therefore, it is recommended to analyze Li-salts with this spectrophotometry method. NO_2_^–^ concentrations in all Li-salts were quantified by ion chromatography (IC) and remained negligible (<1 μmol L^–1^). Ethereal solvents that are stable during Li-NRR, such as tetrahydrofuran, 1,2-dimethoxyethane, and 2-methoxyethyl ether, were screened by IC. Ethanol was also evaluated, since it is often used as a proton source for Li-NRR. None of the organic solvents showed any NO_*x*_^–^-related peak (Figure S13).

## Implications of NO_*x*_ Impurities for the Li-NRR Experimentalists

**Figure 4:**
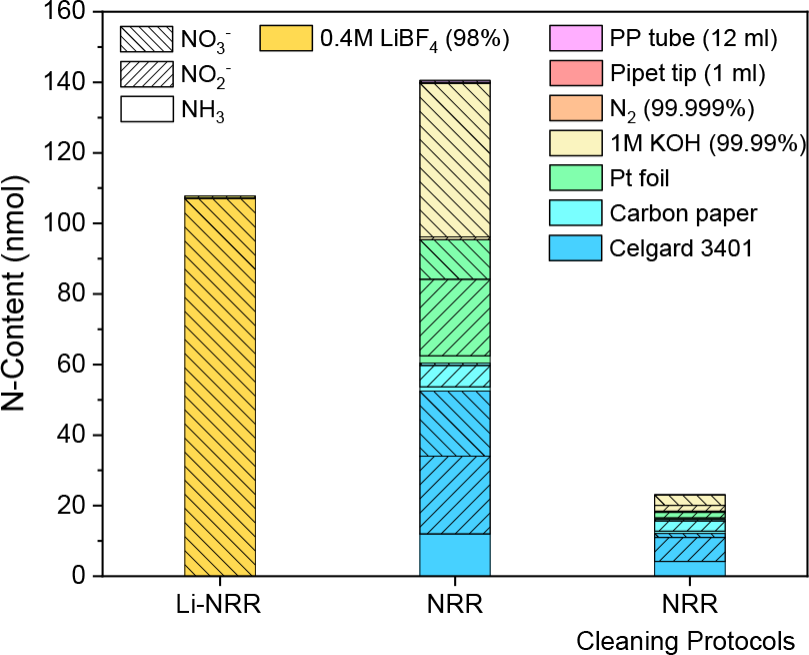
Estimation of the minimum background level of NH_3_, NO_2_^–^, and NO_3_^–^ with and without the most effective cleaning procedures. Values were obtained from Figure S12 and Tables S1–S4, assuming the N_2_ flow (20 mL min^–1^, 99.999%), membrane area (Celgard, 10 cm^2^), working electrode (carbon paper, 1 cm^2^), counter electrode (Pt foil, 4 cm^2^), electrolyte (1 M KOH, 10 mL), 1 pipet tip, and 1 tube with a total experiment time of 15 min. For Li-NRR, only ^14^N_2_ and electrolyte impurities were considered. The applied cleaning procedures for NRR were as follows: alkaline wash for Celgard 3401 membrane and carbon paper, heat treatment for Pt foil, KOH desiccator storage, and rinsing lab consumables with water.

Other extraneous N sources from atmospheric exposure are limited in Li-NRR systems because most handling and storage of solvents, salts, and cell materials are conventionally done in a glovebox, with the main motivation to control moisture content. The content of N contaminations in our feed gases and lab consumables is negligible (except ^15^N_2_), thus only NO_3_^–^ impurities in the Li-salt seem to be relevant for Li-NRR. It is important to note that NO_3_^–^ (most likely present as LiNO_3_) cannot simply be removed by a heat treatment,^45^ since the decomposition temperature of LiNO_3_ (≥500 °C) is much higher than those of LiBF_4_, LiPF_6_, and LiTFSI.^47^ With the hypothetical experimental conditions stated in Figure 4, roughly 107 nmol of NO_3_^–^ can potentially be reduced into NH_3_ during cell operation, leading to a yield of 0.12 nmol s^–1^ cm^–2^. Our estimated NO_3_^–^ content can differ significantly if higher salt concentrations are used or with different Li-salt batches that contain more NO_3_^–^. Nevertheless, it is not realistic to expect that NH_3_ yields obtained by the electroreduction of NO_3_^–^ will approach the recently obtained 2500 nmol s^–1^ cm^–2^ at 1 A cm^–2^,^48^ and 150 nmol s^–1^ cm^–2^ at a current efficiency near unity (at 15–20 bar).^49^ This, however, might not be true when the Li-NRR reports lower NH_3_ yield (e.g., when operating at ∼1 bar). Overall, we find that N impurities are less relevant for the Li-NRR field, although it remains good practice to assess the NO_3_^–^ content in the Li-salts to be certain of the origin of NH_3_.

## Estimation of a Minimum Background Level for Aqueous NRR Measurements

In the NRR, the atmospheric N contributions are more severe, as experiments are generally not performed in a controlled environment, including storage of chemicals and cell materials in ambient air. By combining the most important findings from this study, as illustrated in Figure 4, a background level of ∼140 nmol was estimated. By assuming that most NO_*x*_^–^ species electroreduce into NH_3_, an obtained yield of 0.16 nmol s^–1^ cm^–2^ is already enough for a NRR catalyst to be labeled as plausible.^8^ Approximately 84% of these impurities can be avoided by applying the most effective cleaning procedures. These are material dependent and include alkaline washing for membranes and electrodes, heat treatment for the Pt foil, desiccator storage for salts, and rinsing lab consumables with ultrapure water. Important factors such as catalyst impurities and the influence of gloves are excluded from this analysis because they may vary between studies. Extra care must be taken when validating electrocatalytic NRR activity with ^15^N_2_ gas, since ppm levels of ^15^NH_3_ were detected by our GC-MS and ^15^NO*_x_* by others. Cleaning the feed gases is not straightforward, since our analysis shows that commonly adopted liquid scrubbers do not properly eliminate the NO_*x*_ contaminations, due to limited mass transport and reactivity. More importantly, the trapping efficiency should be evaluated at conditions close to experimental conditions, as we show that factors such as flow rate and duration of the experiment highly affect the removal efficiency. For these reasons we strongly recommend the application of commercial gas purifiers that exhibit the best performance at all relevant conditions. An absolute minimum background level is rather difficult to assess because of the large variety of experimental approaches within the research community. Nevertheless, we provide experimentalists with recommendations and various cleaning procedures in order to reduce the effect of impurities to an acceptable minimum.

## Supporting Information Available

Materials and methods, experimental details, quantification methods and calibration curves, schematic of the setups, electrolyte storage data, ^15^NH_3_ quantification in ^15^N_2_, additional filter performance tests, complete material screening data set, impurities in Li-mediated NRR, supplementary tables, and literature overview of reported N-impurities (PDF)
