# Supplementary material for: Identification, Quantification, and Elimination of NOx and NH3 Impurities for Aqueous and Li-Mediated Nitrogen Reduction Experiments
Source: ACS Energy Lett. 2023 Aug 2;8(8):3614–20. doi: 10.1021/acsenergylett.3c01130 (PMC10425974; doi:10.1021/acsenergylett.3c01130)
Supplement: Supplementary file 1 — nz3c01130_si_001.pdf [file nz3c01130_si_001.pdf]

Supplementary Information for:

Identification, Quantification and Elimination of NO_x and NH_3 impurities for Aqueous and Li-mediated Nitrogen Reduction Experiments

Boaz Izelaar[‡§], Davide Ripepi[†§], Dylan D. van Noordenne[†], Peter Jungbacker[†], Ruud Kortlever[‡*], Fokko M. Mulder[†*]

[‡]Large Scale Energy Storage, Process and Energy Department, Faculty of Mechanical, Maritime and Materials Engineering, Delft University of Technology, 2628 CB Delft, The Netherlands

[†]Materials for Energy Conversion and Storage, Chemical Engineering Department, Faculty of Applied Sciences, Delft University of Technology, 2629 HZ Delft, The Netherlands

Corresponding Authors; E-mail: R.Kortlever@tudelft.nl, F.M.Mulder@tudelft.nl

[§]These authors contributed equally to this work.

Experimental Methods

Materials.

Only ultrapure water (Millipore Milli-Q IQ 7000) was used for cleaning and solution preparation throughout this work. All materials were supplied by Sigma Aldrich unless stated otherwise: $Cu(NO_3)_2{\cdot}2.5H_2O$ (98%, Alfa Aesar), KOH (85% and 99.99%), NH_4Cl (99.99%), KNO_2 (97%), KNO_3 (99%), $NaClO_2$ (25wt% in H_2O), Sulfamic acid (99.3%), HCl (37%), $LiClO_4$ (99.99%), $LiBF_4$ (98% and 99.99%), $LiPF_6$ (98%, Honeywell), LiTFSI (98%), ethanol (anhydrous, VWR), isopropanol (98%, VWR), tetrahydrofuran (99.5%, anhydrous), 1,2-dimethoxyethane (99.5%, anhydrous), 2-methoxyethyl ether (99.5%, anhydrous), 1/8'' HDPE spheres (McMaster), Hydrophobic frit (19.6 mm x 3.2 mm, Biocomma), glass wool – silane treated, anion exchange membrane (Selemion), microporous membrane (Celgard 3401), carbon paper (Alfa Aesar), Pt foil (2.5 cm x 2.5 cm x 0.025 cm, Mateck), pipet tips (Thermo Fischer Scientific), polypropylene sample tubes (1.5 ml Eppendorf, 12 ml Kartell), Latex and Nitrile gloves (Ansell, size M).

Ammonia quantification by GC/GC-MS.

Gaseous ammonia quantification was carried out with gas chromatographic (GC)[1] and gas chromatography-mass spectrometry (GC-MS)[2] methods with a lower detection limit of about 0.15 and 1 ppm, respectively. A TRACE™ 1300 Gas Chromatograph (from Interscience BV - Thermo Fisher Scientific) was equipped with Agilent Select Low Ammonia column located in the GC oven chamber. Once eluted from the chromatography column, the analyte is partitioned between a pulse discharge detector (PDD) and a single quadrupole mass spectrometer (ISQ™ from Thermo Fisher Scientific), which simultaneously analyse the sample with matching retention times. Calibration standards were prepared diluting certified calibration gas mixtures of 13.8 ppm and 2.2 ppm of NH_3 in N_2 with purified N_2. Details of the detection method and calibration curves are available elsewhere.[1, 2]

Gaseous NO_x quantification by the chemiluminescence analyser.

A chemiluminescence 200E Nitric Oxides Analyser (API Teledyne) was used to measure gaseous NO and NO_2. The amount of nitric oxide (NO) present in the sample gas is directly determined from the infrared light ($h\nu_{[1200\,nm]}$) emitted by the reaction between NO and ozone (O_3), as shown in equations S1-S2.

$$NO + O_3 \rightarrow NO_2^* + O_2 \qquad \text{(S1)}$$

$$NO_2^* \rightarrow NO_2 + h\nu_{[1200\,nm]} \qquad \text{(S2)}$$

The emitted light of wavelength 1200 nm is then detected by the photo-multiplier tube light. In addition, NO_2 can be measured as sum of NO and NO_2 present in the gas sample. In this case, the gas sample passes through a molybdenum catalyst held at about 315 °C, where the NO_2 reacts to produce NO gas. Thus, the formed NO (together with the NO already present in the sample) is detected by reaction with ozone and generation of infrared light, following the equations SS1-S2. The total inlet gas flow rate of the gas chemiluminescence nitric oxide analyser was 700 mL min^{-1}, as the instrument requires a relatively high gas flow. The gas purification scrubbers and filters were tested on a flow rate ranging between 1 and 50 mL min^{-1}, and feeding the remaining flow aliquot as He by mean of dedicated mass flow controllers, as illustrated in **Figure S3**. The detection limit of the NO analyser is 1 ppb.
Quantification of NO_x in $^{15}N_2$ was not performed due to the high cost of $^{15}N_2$ and because the NO_x chemiluminescence analyzer requires high flow rates (1 L min^{-1}) in combination with a long equilibration time (> 30 min).

N impurity assessment and removal.

All sample tubes, pipet tips, bottles, glassware (including the commercial and in-house made scrubber), and other involved materials in the sample handling and storage were always excessive prewashed with water. During a typical impurity assessment before and after an applied cleaning procedure, an experimental component with a predefined area (if applicable) was submerged in freshly prepared 0.1 M KOH (often 5 mL) in a sample tube and sonicated for 15 min. Aliquots were withdrawn from the

sample tube for NH_3, NO_2^- and NO_3^- quantification. Four cleaning procedures were evaluated and consisted of; rinsing excessively with water; alkaline wash by submerging the material in 0.1 M KOH followed by 15 min of sonication; thermal decomposition in a tubular furnace (Lenton Blue) at 200 °C (10 °C min^{-1}, 12 hour) under Ar atmosphere; electrochemical NO_x^- reduction by performing 10 cyclic voltammetry scans in between -0.2 V to -0.7 V vs. RHE, where the sample was first rinsed with isopropanol and water as elaborately described in ref [3].

$NH_{3(aq)}$, NO_2^- and NO_3^- spectrophotometric quantification.

The quantification of ammonia was performed by the Berthelot method.[4] A volume of 1.33 mL aliquot (typically 0.1 M KOH) was neutralized by adding 112 μL of 0.5 M H_2SO_4. Both 360 μL of phenol nitroprusside and alkaline hypochlorite (0.2% sodium hypochlorite) were added directly afterwards and stirred on a vortex shaker. An observable colour change ranging from light green to dark blue appeared after 30 min of incubation time. The coloured solutions were transferred to PMMA cuvettes (10 mm x 10 mm x 30 mm) and were inserted in the UV-Vis spectrophotometer (Hach DR6000) for analysis. Six different NH_4Cl concentrations of 0.01, 0.05, 0.1, 0.5, 1 and 2 ppm NH_3 in 0.1 M KOH were prepared to construct the calibration line with the maximum absorbance at 633 nm. The fitted linear curve with $A = 0.7279C_{NH3} - 0.001$ and $R^2 = 0.9999$ showed decent reproducibility (**Figure S14**).

Aliquots that interfered with the NO_2^- peak in the ion chromatogram were quantified with the spectrophotometric Griess test. A commercially available Griess reagent mixture with a detection range between 0.007-3.28 ppm NO_2^- (Spectroquant, Merck) was used. 2 ml Aliquots of 0.1 M KOH solutions were neutralized with 0.5 M H_2SO_4. Approximately, 30 mg of the Griess reagent mixture was added to the neutralized aliquots and mixed thoroughly. After 10 min of incubation time the solution appears between light pink and dark magenta ranging from low to high concentration. Five different concentrations from KNO_2^- of 0.02, 0.05, 0.1, 0.5, 1 ppm NO_2^- were used to construct the calibration line by taking the maximum absorbance at 542 nm. The calibration line was perfectly linear with $A = 0.8071C_{NO2-} - 0.0001$ and $R^2 = 1$ (**Figure S15**). The spectrophotometric samples were always compared versus a fresh 0.1 M KOH solution as blank.

Li-salts commonly used in Li-NRR were screened by dual-wavelength UV spectroscopy for NO_3^- detection. UV absorbance of NO_3^- in water can be detected at 210 nm, which is the same wavelength as for other organic compounds, such as carbonates. To compensate for this, the UV absorbance at 270 nm (common wavelength for most organics) was substracted by $A_{NO3-} = A_{210} – 2A_{270}$. Additionally, small quantities of HCl and sulfamic acid were added to reduce carbonate and nitrite interference, respectively. For the sample preparation, 2.5 mL aliquots were mixed with 50 μL 1 M HCl and 50 μL 10.5 mM sulfamic acid. Subsequently, the mixture was transferred to 10 mm Quartz glass cuvettes (Hellma). Five different KNO_3^- concentrations of 0.05, 0.1, 0.25, 1, 2 ppm NO_3^- were prepared in water to construct a linear calibration line (**Figure S16**) with $A_{NO3-} = 0.1126\ C_{NO3-} - 0.0006$ and $R^2 = 0.9998$.

NO_2^- and NO_3^- quantification by ion chromatography.

Nitrite and nitrate were determined through ion chromatography (IC). The IC tests were performed by an Thermo Fischer Scientific, Dionex, Integrion HPIC System, equipped with a conductivity detector and AS18-Fast anion column. Additionally, according to application note 72481 from Thermo Fischer Scientific,[5] AutoNeutralization was installed to remove the KOH background. This removes the need for dilution of samples before injection and thus increases accuracy. With the used setup, a sample is manually injected, filling the sample loop (25 μL). Milli-Q water delivered from the external AXP pump and trap column (Dionex IonPac ATC-HC 500 trap column) is used to transfer the sample from the sample loop at 0.5 mL min^{-1} to the neutralizing suppressor and collected on the concentrator column. As the sample passes through the suppressor (4 mm high capacity Dionex AERS 500 Anion Electrolytically Regenerated Suppressor), the cation (K^+ in our case) is exchanged with hydronium thereby neutralising the alkaline sample. The anions of interest are retained on the concentrator column while the water flows to waste, thereby also concentrating the trace anions of the sample. The anions of interest elute from the concentrator column to the guard column and separation column. Here the anions are separated using an eluent consisting of 21.8 mM KOH in milli-Q water at 0.25 mL min^{-1} on the Dionex AS18-Fast anion column. As the analyte peaks elute from the column, they are detected by

suppressed conductivity detection using a 2 mm Dionex AERS 500 Anion Electrolytically Regenerated Suppressor and conductivity detector. Two calibration lines were constructed by preparing five different concentrations of 0.01, 0.05, 0.25, 0.5 and 1 ppm NO_2^- and NO_3^- in H_2O from KNO_2 and KNO_3, respectively. **Figure S17** indicates a clear peak separation between different anion species, which allows accurate integration of the respective peaks. The integrated conductance vs. NO_2^- and NO_3^- concentration were plotted in **Figure S18**, which show a linear relationship with $I = 0.7476*C_{NO_2^-} - 0.0082$ ($R^2 = 0.998$) and $I = 0.5821*C_{NO_3^-} - 0.0067$ ($R^2 = 0.997$).

Low concentrations of Li-salts (0.1 M) were injected into the IC only for NO_2^- screening because anions such as ClO_4^- and BF_4^- interferes with the NO_3^- peak (**Figure S19**). In case of $LiBF_4$ (98%), an unknown compound also interfered with the NO_2^- peak, which made it difficult to accurately determine the concentration. Higher salt concentrations (> 0.1 M) were not considered because it inflates the interference effects.

Supplementary Figures

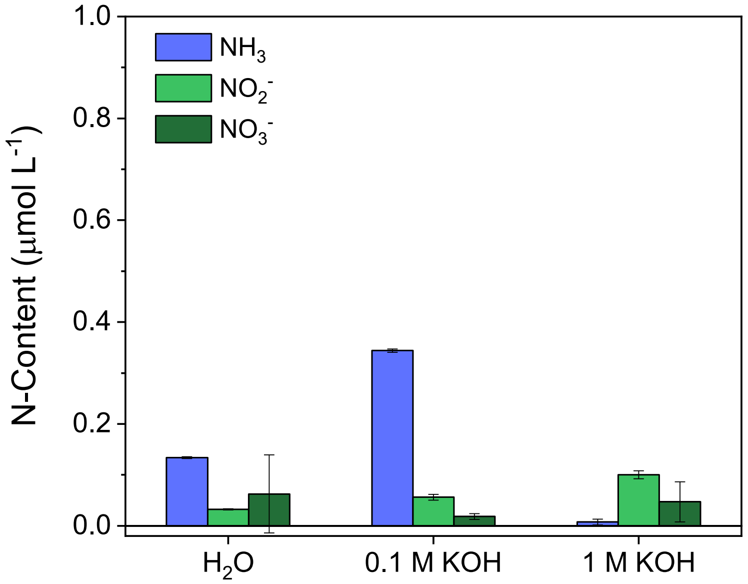

Figure S1. 24 hour open-to-air exposure of 2 ml H_2O, 0.1 M KOH and 1 M KOH in a 12 ml sample tube. The presented values are corrected with blank samples measured at t = 0. Error bar indicates the standard deviation of triplicates.

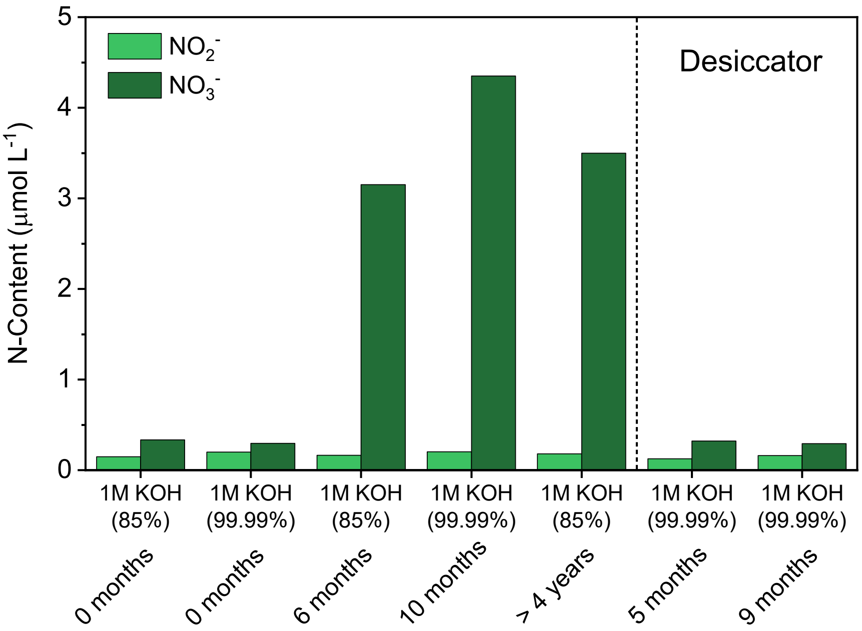

Figure S2. Comparison between KOH salt storage conditions in a chemical safety cabinet and vacuum desiccator. The projected impurity concentrations were quantified by IC using freshly prepared 1 M KOH solutions.

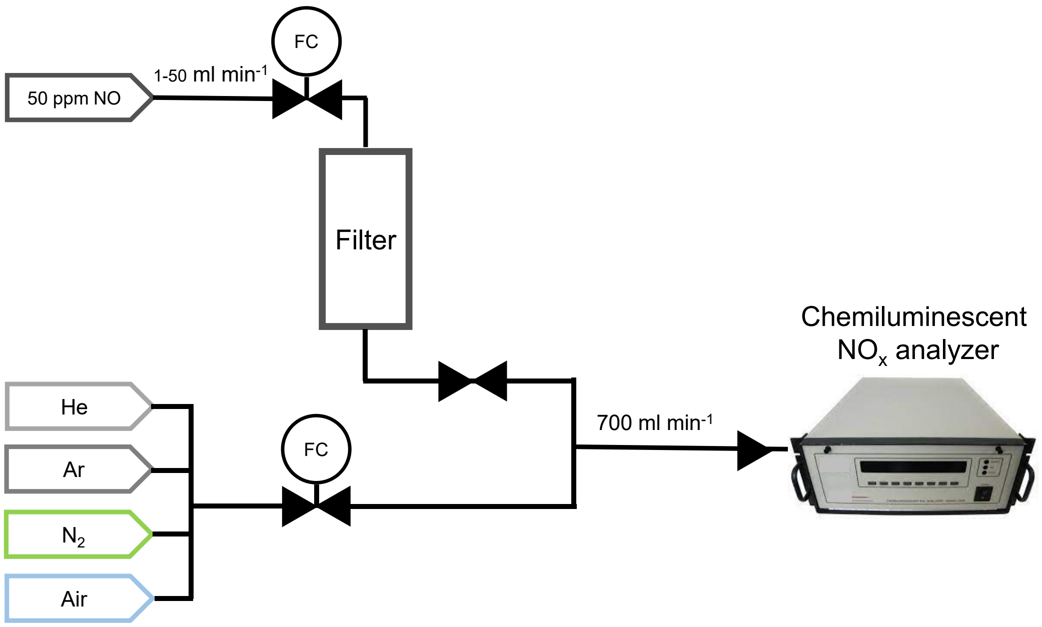

Figure S3. Schematic of the NO_x quantification measurements.

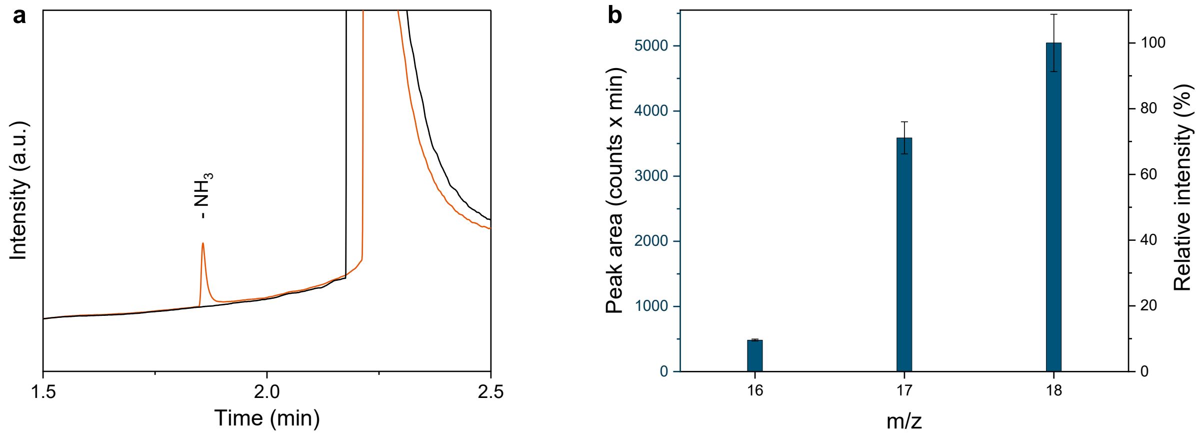

Figure S4. (a) Close up of the chromatograph around the ammonia elution time of the GC from the analysis of $^{15}N_2$ gas. Ammonia contaminations (9.8 ppm) are detected in the $^{15}N_2$ gas directly connected to the GC inlet (orange line). Interestingly, when the $^{15}N_2$ gas was dosed via a mass flow controller (not passivated against ammonia adsorption), no ammonia was detected (black line). (b) Integrated peak areas of the mass-to-charge ratio (m/z) of the ions detected with GC-MS at 1.84 min retention time (i.e. ammonia retention time). The relative intensity of the m/z corresponds to the $^{15}NH_3$ ionization fragments. Error bars correspond to the standard deviation of three independent measurements.

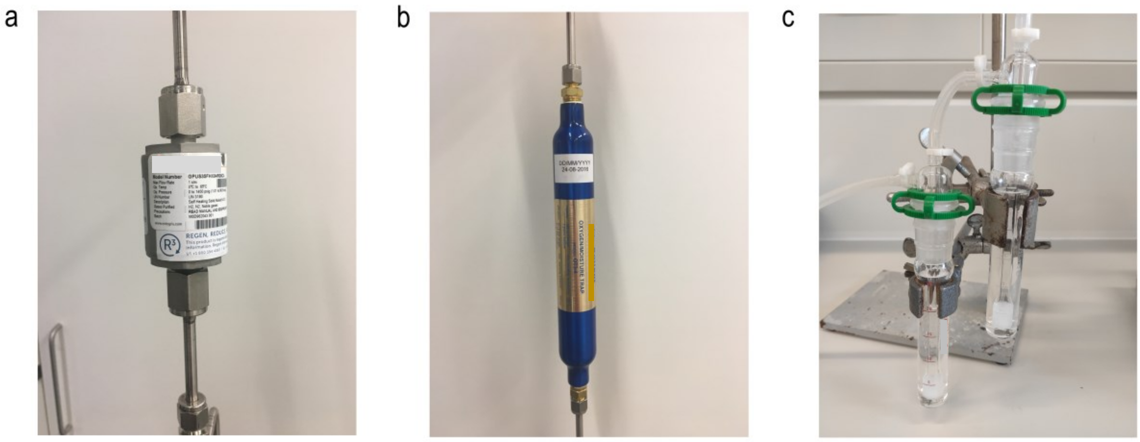

Figure S5. Photographs of the tested gas filters. (a) Entegris GPUS35FHX and (b) Agilent OT3-4 commercial packed gas filters. (c) Two 20 mL scrubbers (Supelco Analytical, 6-4835) with liquid trap solution connected in series.

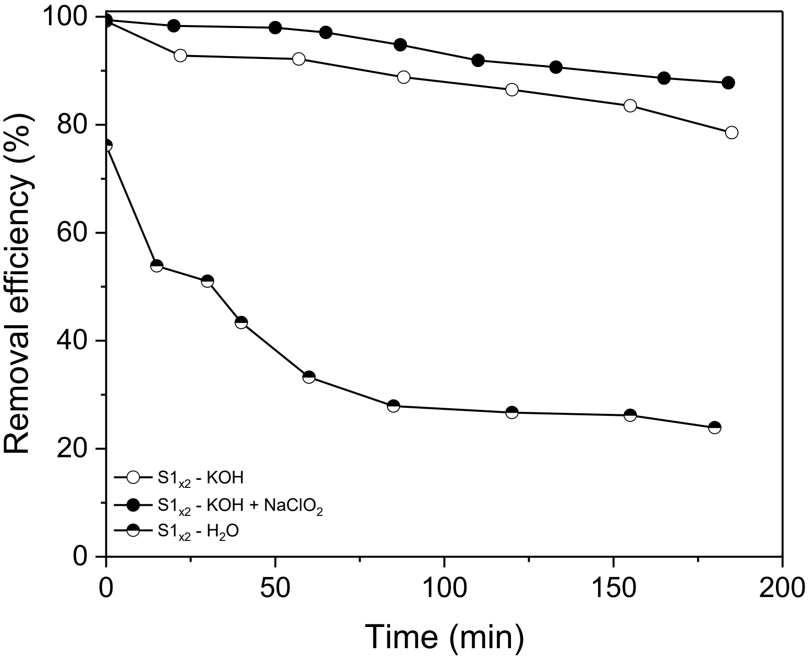

Figure S6. NO_x removal efficiency over time, measured at 10 mL min^{-1} of 50 ppm NO in He for two 20 mL scrubbers (Supelco Analytical, 6-4835) connected in series containing MilliQ water (half-filled symbols), 0.1 M KOH (open symbols) and 0.1 M KOH + 0.1 M $NaClO_2$ (solid symbols) as with liquid trap solution.

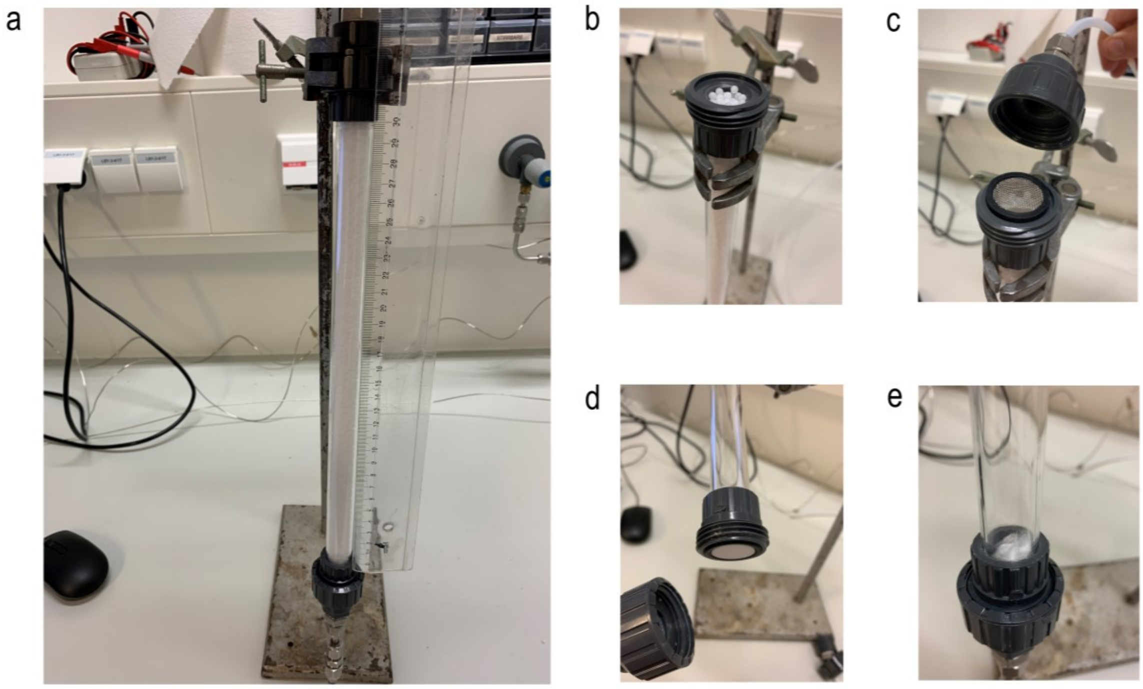

Figure S7. Photographs of the in-house made scrubber. (a) Assembled scrubber with 30 cm length and 1.5 cm diameter made of polymethyl-methacrylate. (b) Visible inert 1/8'' HDPE beads as packing material to improve the tortuosity were filled from the top part prior to each experiment, (c) and closed off with a stainless steel mesh to keep the beads in place during operation. (d) A hydrophobic frit (19.6 x 3.2 mm) was inserted at the bottom of the column (e) with an additional layer of glass wool.

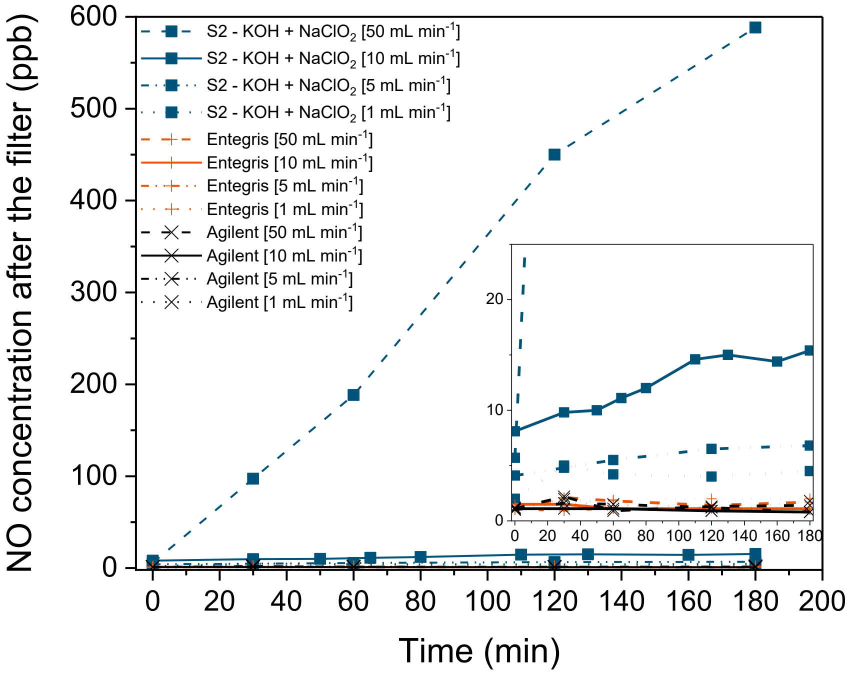

Figure S8. NO concentration measured over time at the outlet of each gas filter tested with an inlet gas mixture of 50 ppm NO in He at different flow rates (50 mL min^{-1} dashed line, 10 mL min^{-1} solid line, 5 mL min^{-1} dash-dot line, and 1 mL min^{-1} dotted line). In-house made scrubber (S2) filled with a 0.1 M KOH and 0.1 M $NaClO_2$ trapping solution is shown in blue, while the commercial Entegris and Agilent packed filters are shown in orange and black, respectively.

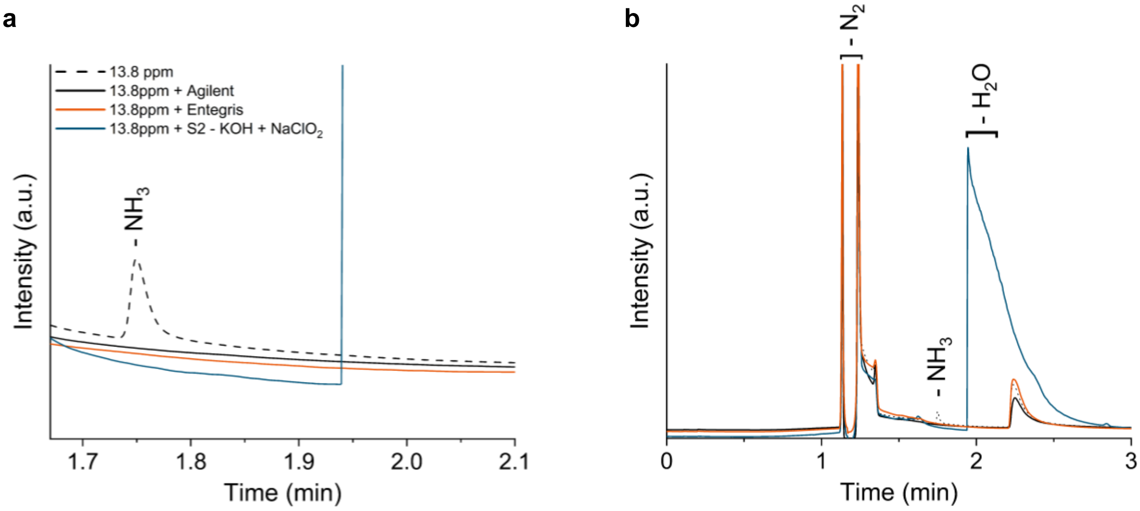

Figure S9. (a) Close up of the chromatograph around the ammonia elution time obtained from the analysis of a 13.8 ppm NH_3 in N_2 calibration gas flowing at 10 mL min^{-1} connected directly to the GC (dashed black line) and purified with the Agilent OT3-4 filter (solid black line), Entegris GPUS35FHX filter (solid orange line) and in-house made scrubber (S2) filled with a 0.1 M KOH and 0.1 M $NaClO_2$ trapping solution (solid blue line) prior entering the GC. All the ammonia contained in the gaseous analyte is captured by the filters. (b) Full chromatograph highlighting that the purification with the in-house made scrubber introduces a significant amount of water into the feed gas.

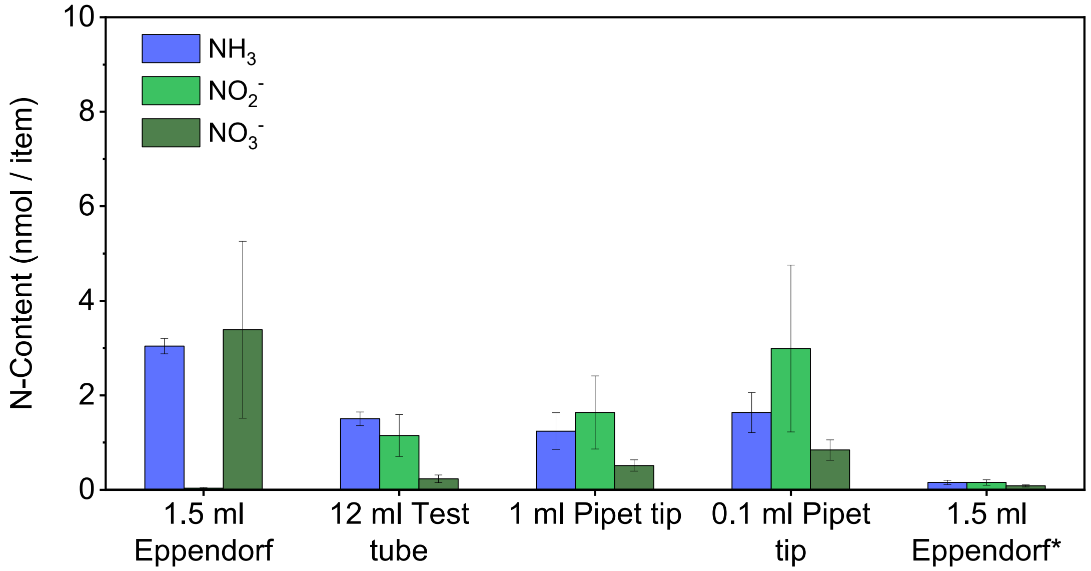

Figure S10. Screening of various lab consumables stocked in our laboratory. 1.5 ml and 12 ml polypropylene sample tubes were completely filled with 0.1 M KOH. The 0.1 ml and 1 ml polypropylene pipet tips were transferred into a pre-cleaned sample tube filled with 12 ml and 6 ml of 0.1 M KOH, respectively. All consumables were sonicated for 15 min. This procedure was repeated 5 times using the same solution. *Directly analysed after arrival.

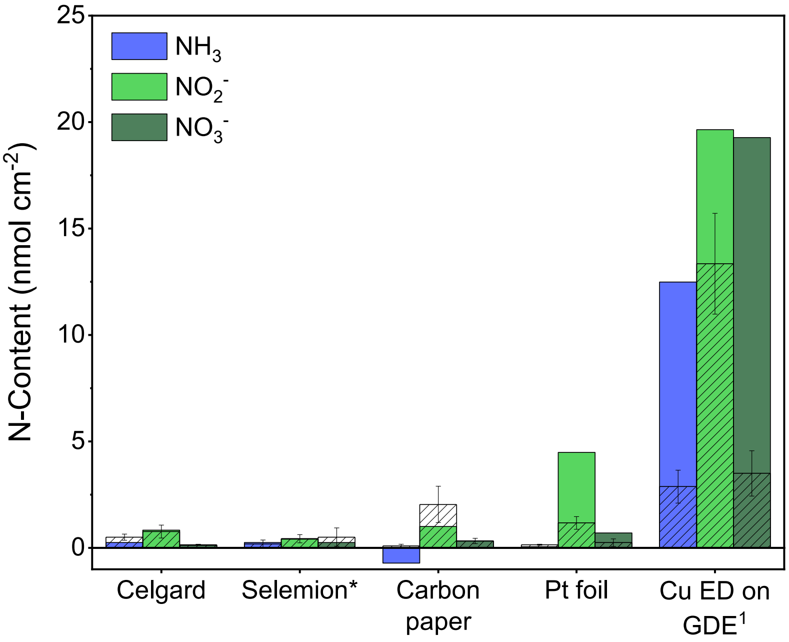

Figure S11. Release of N impurities after 1 hour sonication in 0.1 M KOH after the following pre-treatment steps; 15 min of ultra-sonication in 0.1 M KOH; [1] rinsed with H_2O and isopropanol, than electrochemically reduced by performing 10 cyclic voltammetry scans with in a reduction regime (-0.2 V to -0.7 V vs. RHE) and rinsed with H_2O afterwards as elaborately described in ref [3].* NO_2^- was quantified with the spectrophotometric Griess test due to identified Cl^- overlap in the ion chromatogram.

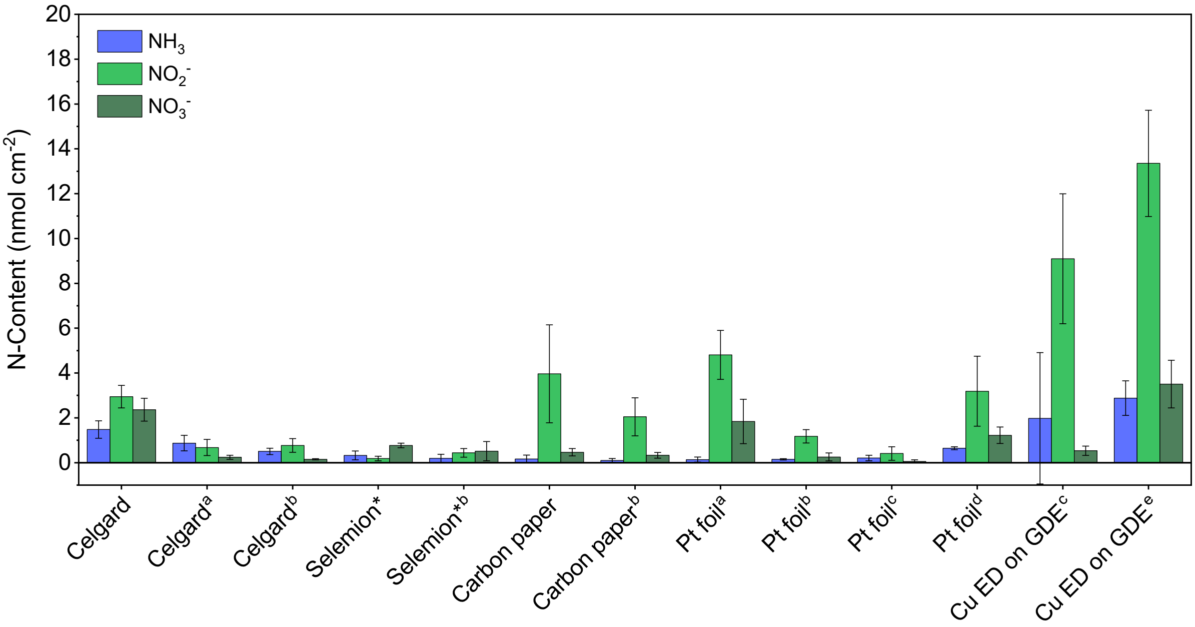

Figure S12. Screening of typical electrochemical cell components and the effectiveness of various cleaning procedures. Obtained concentrations of NH_3, NO_2^- and NO_3^- were converted to nmol and normalized by the geometric area. All components, except the carbon paper had a 2.5 cm x 2.5 cm dimension. The carbon paper and Cu GDE were finely cutted discs with a diameter of 1.2 cm. The cell materials were ultra-sonicated in 5 ml of 0.1 M KOH for 15 min with different pre-treatment steps. Indicated as unlabelled is untreated and fetched from the as received package. (a) rinsed excessively with H_2O, (b) sonicated for 15 min in 0.1 M KOH, (c) thermal decomposition in Ar at 200 °C overnight (12 hours), (d) sonicated for 15 min in 0.1 M KOH plus flame annealing with butane flame torch, (e) rinsed with H_2O and isopropanol, than electrochemically reduced by performing 10 cyclic voltammetry scans with only reductive currents (-0.2 V to -0.7 V vs. RHE) and rinsed with H_2O afterwards as elaborately described in ref [3]. * NO_2^- assay performed with the spectrophotometric Griess test due to identified Cl^- overlap with NO_2^- in the ion chromatogram. Every component is measured in triplicates.

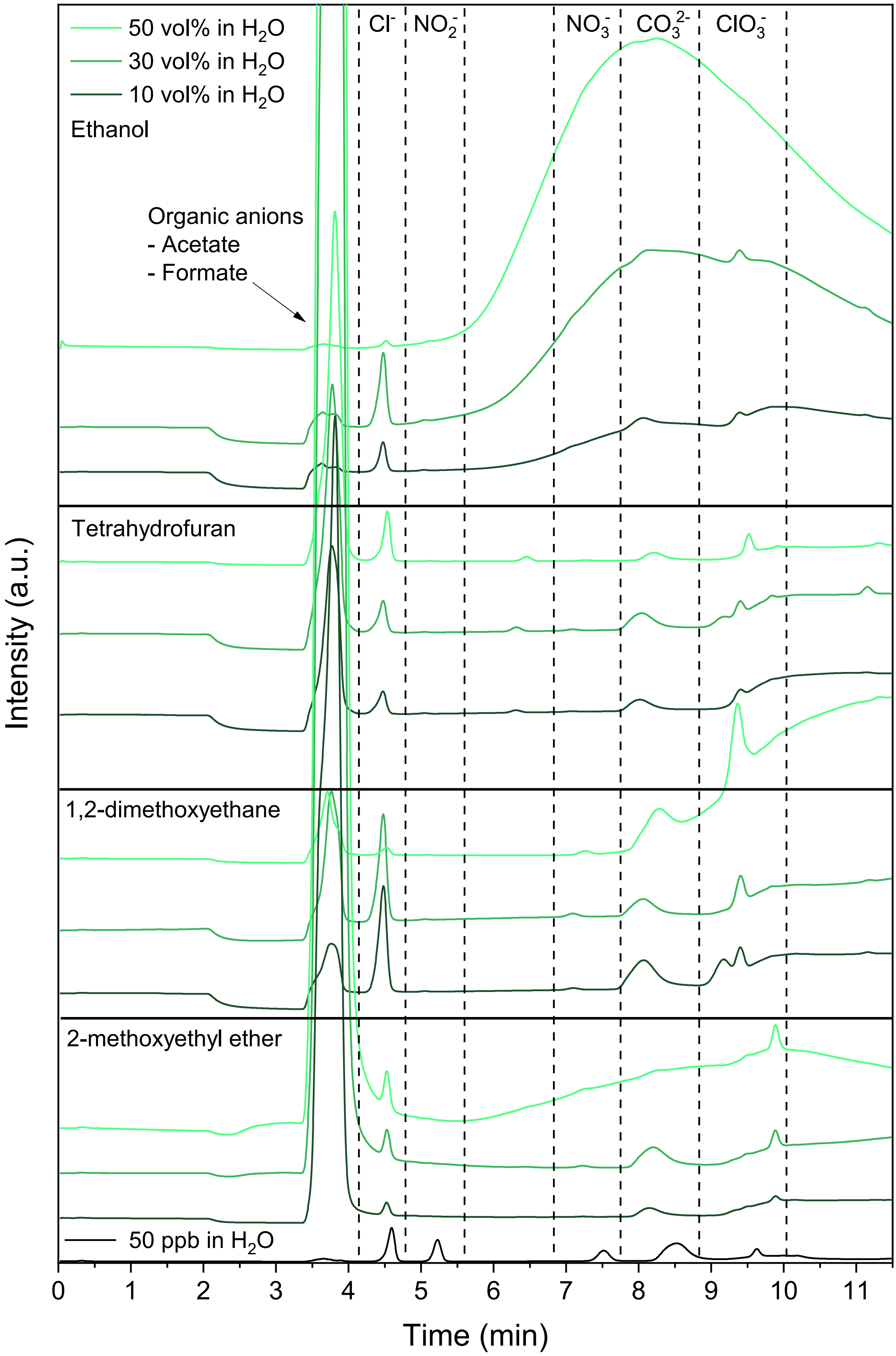

Figure S13. Ion chromatograms of 10, 30 and 50 vol% of ethanol, tetrahydrofuran, 1,2-dimethoxyethane, 2-methoxyethane in H_2O. An extra ion chromatogram of 50 ppb NO_2^- and NO_3^- in H_2O is plotted as reference. Organic anions, chloride, carbonate and chlorate were also identified by using reference data.[6]

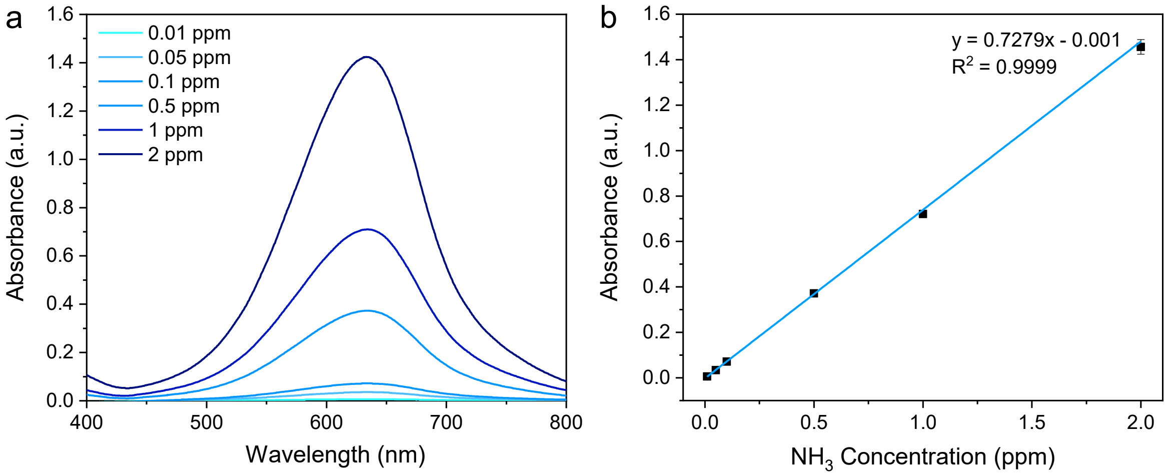

Figure S14. (a) UV-Vis spectra of different NH_3 concentrations in 0.1 M KOH. (b) Fitted calibration curve (blue) from the absorbance at 633 nm versus different NH_3 concentrations.

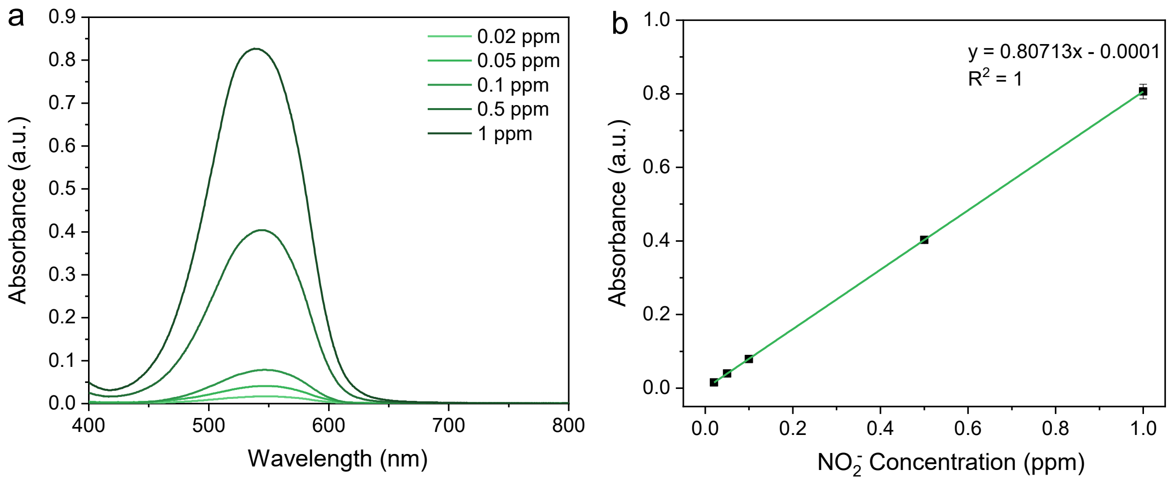

Figure S15. (a) UV-Vis spectra of different NO_2^- concentrations in 0.1 M KOH. (b) Fitted calibration curve (light green) from the absorbance at 542 nm versus different NO_2^- concentrations.

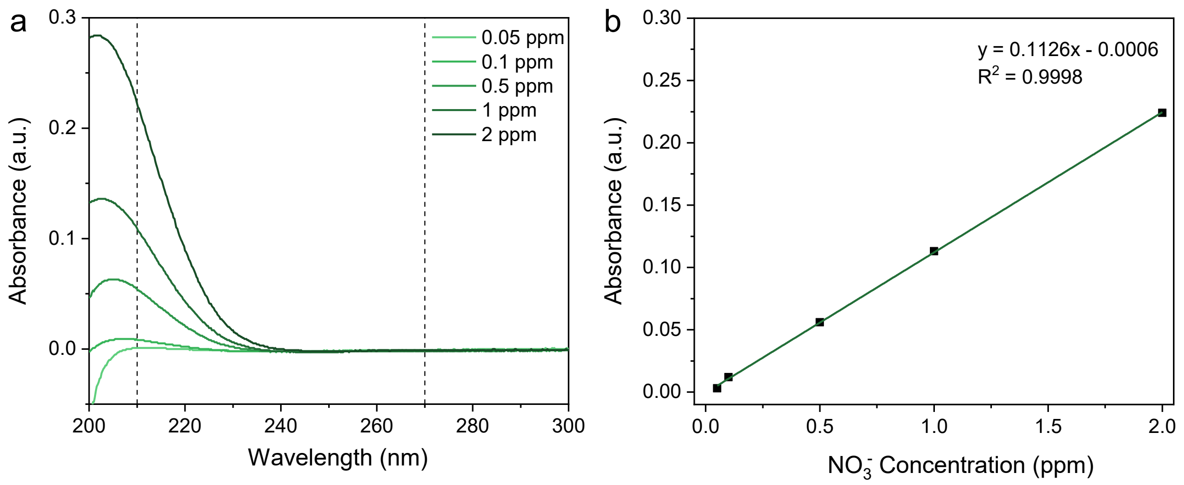

Figure S16. (a) UV spectra of different NO_3^- concentrations in H_2O. (b) Fitted calibration curve (dark green) from the absorbance at 210 nm versus different NO_3^- concentrations.

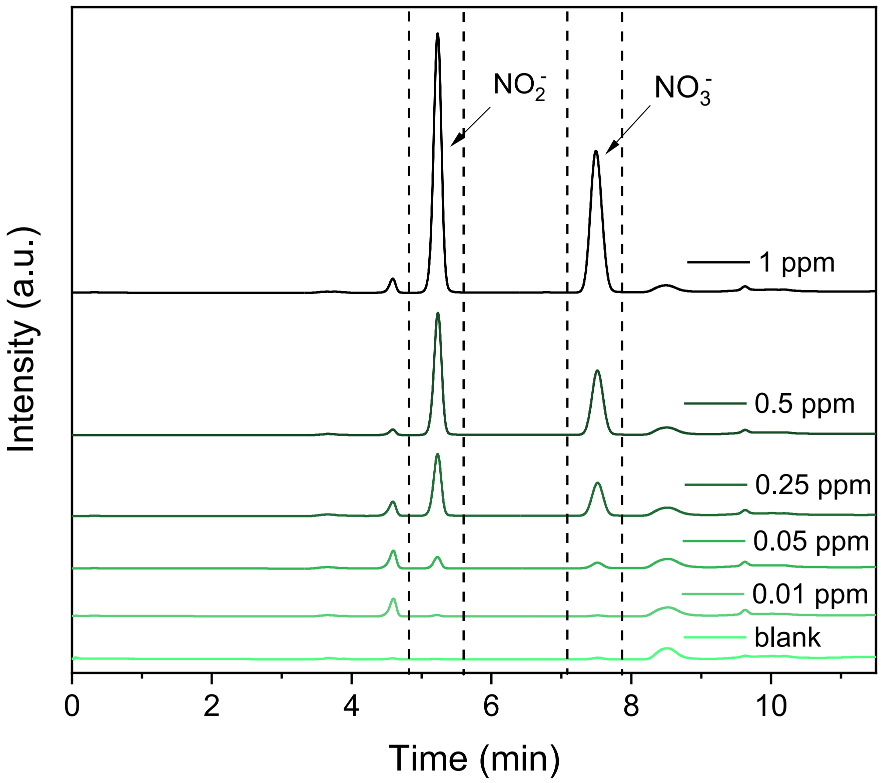

Figure S17. Ion chromatograms recorded at different NO_2^- and NO_3^- concentrations in H_2O.

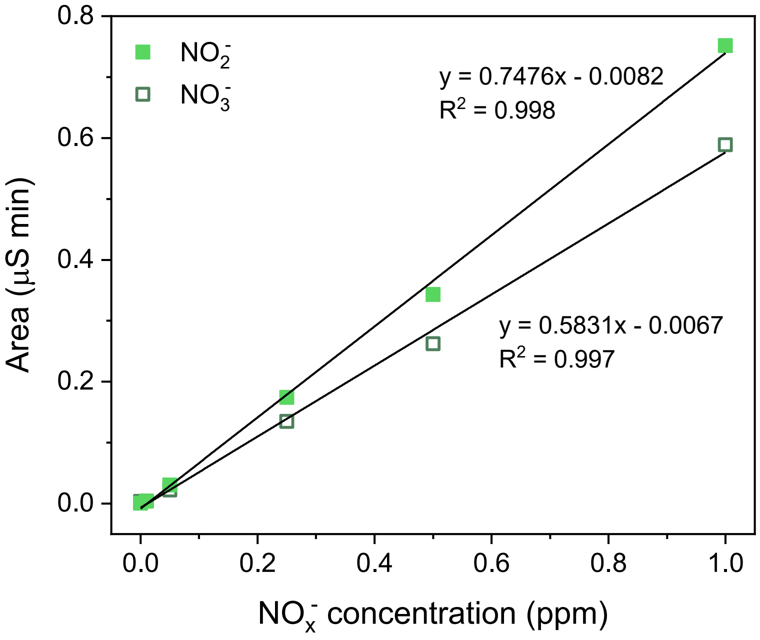

Figure S18. Ion chromatography calibration curves for NO_2^- and NO_3^- in H_2O.

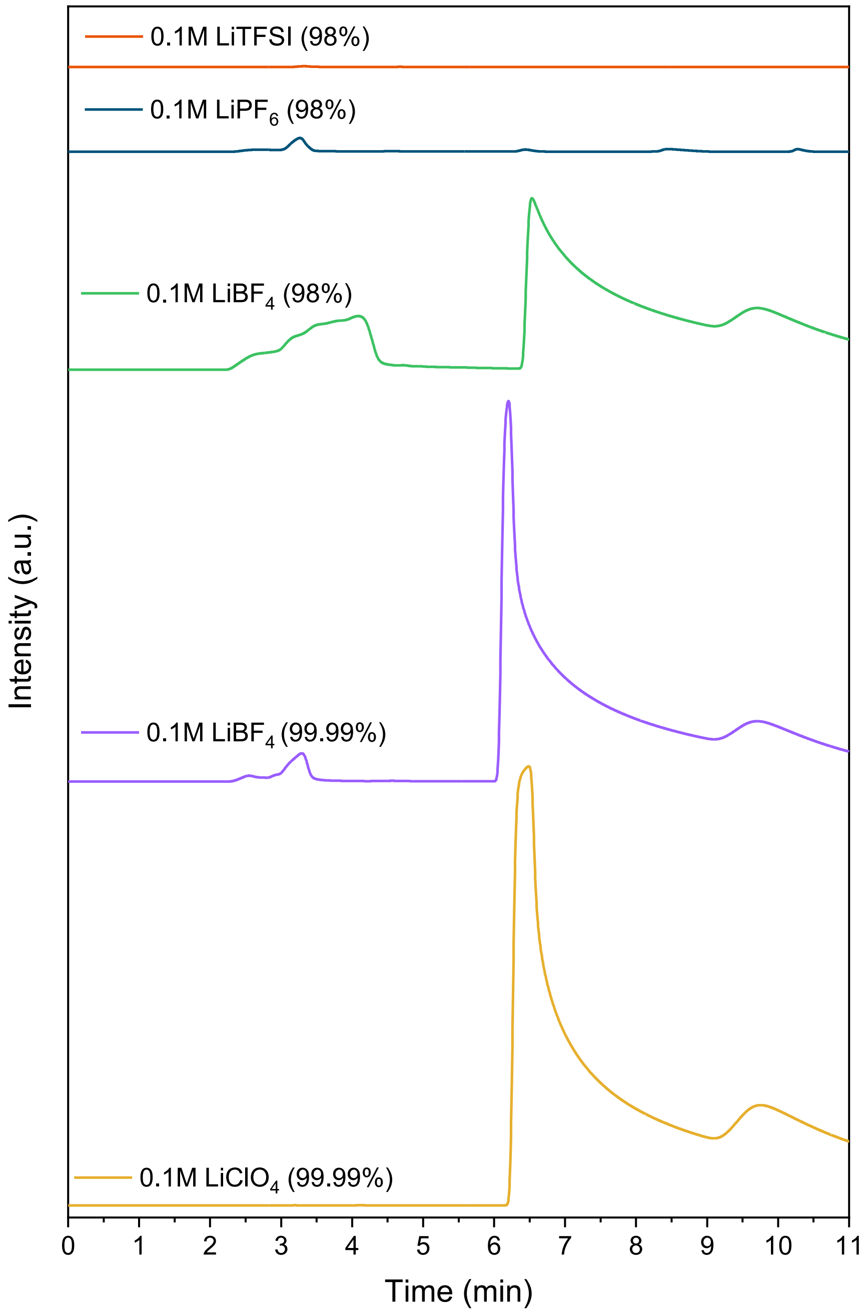

Figure S19. Ion chromatograms of diluted Li-salts concentrations in water.

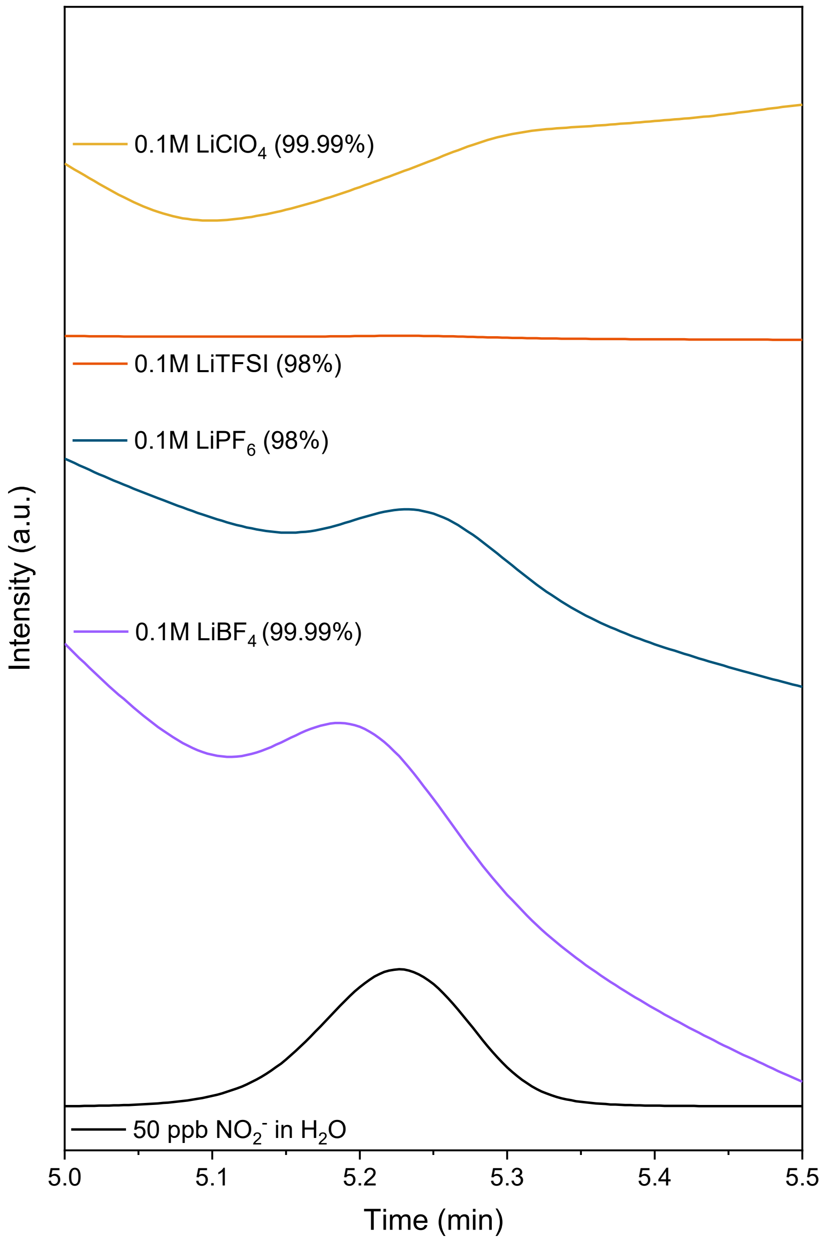

Figure S20. NO_2^- peaks in the ion chromatograms of various diluted Li-salts in H_2O and 50 ppb NO_2^- in H_2O.

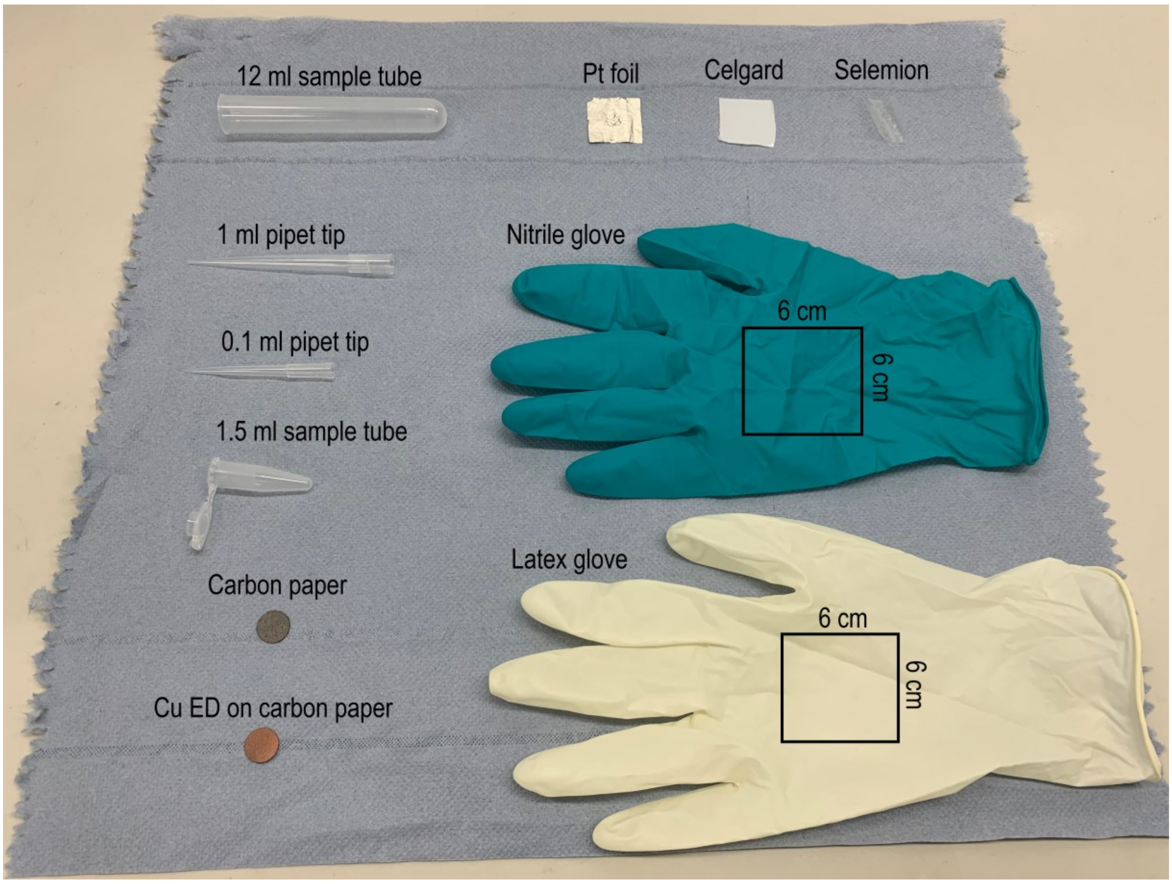

Figure S21. Photographic overview of the lab consumables and components used for screening.

Supplementary tables

Table S1. Data summary of feed gas and ambient impurities measured in the present study and complemented by literature values.

Compounds	NH_3 (ppm)	NO_x (ppm)	Ref
Ambient air	0.003-0.022	0.015-0.04	7
		0-0.0048	8
		0.0015-0.027	This work
Human breath	0.03-3		9
	0.28-1.4		10
He (99.999%)	<0.15	0.0011	This work
$^{14}N_2$ (99.999%)	<0.15	0.0019	This work
		0.0031	8
$^{15}N_2$ (99%)	9.8 ($^{15}NH_3$)		This work
	0–1.61	0–1.03	11
Ar (99.999%)	<0.15	0.0018	This work
		0.0013	8

Table S2. Data summary of impurities in lab consumables measured in the present study and complemented by literature values.

Lab consumables	NH_3^+ (nmol / item)	NO_2^- (nmol / item)	NO_3^- (nmol / item)	Ref
15 ml tube (borosilicate)		0.007	0.66	12
15 ml tube (soda lime)		0.003	1.42	12
15 ml tube (polypropylene)		0.002	0.56	12
12 ml tube (polypropylene)	1.5	1.15	0.23	This work
1.5 ml tube (polypropylene)	0.16 - 3.04	0.035 - 0.16	0.08 - 3.39	This work
1 ml pipet tip (polypropylene)		0 - 0.003	0.011 - 0.11	12
	1.24	1.64	0.52	This work
0.2 ml pipet tip (polypropylene)		0.0008	0.005	12
0.1 ml pipet tip (polypropylene)	1.65	2.99	0.84	This work
Latex gloves		0-1500	0-152000 (full glove)	13
	569.4	10.7	10168 (finger tips)	This work
	182.5	3.35	297 (patch 6x6 cm)	This work
Nitrile gloves	276.2	20.3	9141 (finger tips)	This work
	132.5	10.2	850 (patch 6x6 cm)	
	1644 (patch 5 cm^2)			9

Table S3. Data summary of impurities in various cell components and materials measured in the present study and complemented by literature values.

Materials	NH_3 (nmol cm^{-2})	NO_2^- (nmol cm^{-2})	NO_3^- (nmol cm^{-2})	Ref
Nafion	1 - 25			[9, 14, 15]
Selemion	0.3	0.2	0.8	
Celgard 3401	1.5	2.9	2.3	This work<br>This work
Carbon paper	0.2	4	0.5	This work
Pt foil	0.14	4.8	1.8	This work
Cu electrodeposited on carbon paper (Prepared with $CuNO_3$)	4.8	59	1499	This work
Fe_2O_3		0	41.7 nmol mg^{-1}	[16]
Bi_2O_3		23.5 nmol mg^{-1}	92.3 nmol mg^{-1}	
Al_2O_3		1.98 nmol mg^{-1}	47.6 nmol mg^{-1}	

Table S4. Data summary of impurities in electrolytes and organic solvents measured in the present study and complemented by literature values.

Electrolytes and solvents	NO_2^- (µmol L^{-1})	NO_3^- (µmol L^{-1})	Ref
1 M KOH (85%, Sigma)	0.147	0.333	This work
1 M KOH (99.99%, Sigma)	0.198	0.297	
0.5 M Li_2SO_4	0.713	0 – 180.5	[17]
0.5 M $LiClO_4$		22.4 – 38.4	
0.1 M $LiClO_4$ (99.99%, Sigma)	0.485	0*	This work
0.4 M $LiClO_4$		0*	
0.1 M $LiBF_4$ (98%, Sigma)		4.53	
0.4 M $LiBF_4$		10.69	
0.1 M $LiBF_4$ (99.99%, Sigma)	0.776	1.385	
0.4 M $LiBF_4$		6.25	
0.1 M $LiPF_6$ (98%, Honeywell)	0.385	0*	
0.4 M $LiPF_6$		0*	
0.1 M LiTFSI (98%, Sigma)	0*	0*	
0.4 M LiTFSI		1.23	
10vol% ethanol (anhydrous, VWR)	0.016	0.043	This work
50vol% ethanol	0.009		
10vol% Tetrahydrofuran (anhydrous, 99.5%, Sigma)	0.012	0.172	
50vol% Tetrahydrofuran	0*	0.024	
10vol% 1,2-dimethoxyethane (anhydrous, 99.5%, Sigma)	0.009	0.091	
50vol% 1,2-dimethoxyethane	0	0.177	
10vol% 2-methoxyethyl ether (anhydrous, 99.5%, Sigma)	0*	0.204	
50vol% 2-methoxyethyl ether	0*	0.266	

*Lower than the background, therefore assumed to be 0.

Supplementary references

(1) Zaffaroni, R.; Ripepi, D.; Middelkoop, J.; Mulder, F. M. Gas Chromatographic Method for In Situ Ammonia Quantification at Parts per Billion Levels. *ACS Energy Letters* **2020**, *5* (12), 3773-3777. DOI: 10.1021/acsenergylett.0c02219.
(2) Ripepi, D.; Zaffaroni, R.; Kolen, M.; Middelkoop, J.; Mulder, F. M. Operando isotope selective ammonia quantification in nitrogen reduction studies via gas chromatography-mass spectrometry. *Sustainable Energy & Fuels* **2022**, *6* (8), 1945-1949, 10.1039/D2SE00123C. DOI: 10.1039/D2SE00123C.
(3) Kani, N. C.; Prajapati, A.; Collins, B. A.; Goodpaster, J. D.; Singh, M. R. Competing Effects of pH, Cation Identity, H 2 O Saturation, and N 2 Concentration on the Activity and Selectivity of Electrochemical Reduction of N 2 to NH 3 on Electrodeposited Cu at Ambient Conditions. *ACS Catalysis* **2020**, *10*, 14592-14603. DOI: 10.1021/acscatal.0c04864.
(4) Weatherburn, M. W. Phenol-Hypochlorite Reaction for Determination of Ammonia. *Analytical Chemistry* **1967**, *39* (8), 971-974. DOI: 10.1021/ac60252a045.
(5) ThermoFisher. *Application Notes: 72481 IC trace anions concentrated bases*. https://assets.thermofisher.com/TFS-Assets/CMD/Application-Notes/an-72481-ic-trace-anions-concentrated-bases-an72481-en.pdf (accessed 2022-20-8).
(6) ThermoScientific. Errata - Product Manual for Dionex IonPac AS18 and AG18 Columns. 2012.
(7) RIVM. *luchtmeetnet - NH3 and NO2*. 2014. https://www.luchtmeetnet.nl/componenten (accessed 2022-06-07).
(8) Yu, W.; Buabthong, P.; Read, C. G.; Dalleska, N. F.; Lewis, N. S.; Lewerenz, H.-J.; Gray, H. B.; Brinkert, K. Cathodic NH 4 + leaching of nitrogen impurities in CoMo thin-film electrodes in aqueous acidic solutions †. *Sustainable energy & fuels* **2020**, *4*, 5080-5087. DOI: 10.1039/d0se00674b.
(9) Andersen, S. Z.; Čolić, V.; Yang, S.; Schwalbe, J. A.; Nielander, A. C.; McEnaney, J. M.; Enemark-Rasmussen, K.; Baker, J. G.; Singh, A. R.; Rohr, B. A.; et al. A rigorous electrochemical ammonia synthesis protocol with quantitative isotope measurements. *Nature* **2019**, *570* (7762), 504-508. DOI: 10.1038/s41586-019-1260-x.
(10) Larson, T. V.; Covert, D. S.; Frank, R.; Charlson, R. J. Ammonia in the human airways: neutralization of inspired acid sulfate aerosols. *Science* **1977**, *197* (4299), 161-163.
(11) Dabundo, R.; Lehmann, M. F.; Treibergs, L.; Tobias, C. R.; Altabet, M. A.; Moisander, P. H.; Granger, J. The Contamination of Commercial 15N2 Gas Stocks with 15N–Labeled Nitrate and Ammonium and Consequences for Nitrogen Fixation Measurements. *PLOS ONE* **2014**, *9* (10), e110335-e110335. DOI: 10.1371/JOURNAL.PONE.0110335.
(12) Ishibashi, T.; Himeno, M.; Imaizumi, N.; Maejima, K.; Nakano, S.; Uchida, K.; Yoshida, J.; Nishio, M. NOx Contamination in Laboratory Ware and Effect of Countermeasures. *Nitric Oxide* **2000**, *4* (5), 516-525. DOI: 10.1006/NIOX.2000.0302.
(13) Makela, S.; Yazdanpanah, M.; Adatia, I.; Ellis, G. Disposable Surgical Gloves and Pasteur (Transfer) Pipettes as Potential Sources of Contamination in Nitrite and Nitrate Assays. *Clinical Chemistry* **1997**, *43* (12), 2418-2420. DOI: 10.1093/CLINCHEM/43.12.2418.
(14) Hanifpour, F.; Sveinbjörnsson, A.; Canales, C. P.; Skúlason, E.; Flosadóttir, H. D. Preparation of Nafion Membranes for Reproducible Ammonia Quantification in Nitrogen Reduction Reaction Experiments. *Angewandte Chemie International Edition* **2020**, *59* (51), 22938-22942. DOI: 10.1002/anie.202007998.
(15) Liu, H.; Zhang, Y.; Luo, J. The removal of inevitable NOx species in catalysts and the selection of appropriate membrane for measuring electrocatalytic ammonia synthesis accurately. *Journal of Energy Chemistry* **2020**, *49* (x), 51-58. DOI: 10.1016/j.jechem.2020.01.029.
(16) Chen, Y.; Liu, H.; Ha, N.; Licht, S.; Gu, S.; Li, W. Revealing nitrogen-containing species in commercial catalysts used for ammonia electrosynthesis. *Nature Catalysis* **2020**, *3* (12), 1055-1061. DOI: 10.1038/s41929-020-00527-4.
(17) Li, L.; Tang, C.; Yao, D.; Zheng, Y.; Qiao, S.-Z. Electrochemical Nitrogen Reduction: Identification and Elimination of Contamination in Electrolyte. *ACS Energy Letters* **2019**, *4* (9), 2111-2116. DOI: 10.1021/acsenergylett.9b01573.
